# Light spectrum effects on huperzine A and B accumulation in axenic gametophyte cultures of *Huperzia selago* (L.) Bernh. ex Schrank et Mart

**DOI:** 10.3389/fpls.2026.1933008

**Published:** 2026-10-05

**Authors:** Wojciech J. Szypuła, Marta Maciejczyk, Beata Wileńska

**Affiliations:** Department of Pharmaceutical Biology, Faculty of Pharmacy, Medical University of Warsaw, Warsaw, Poland

**Keywords:** axenic gametophytes, *Huperzia selago*, huperzine A, huperzine B, *in vitro* culture, light spectrum, specialized metabolism, AChE inhibition

## Abstract

**Introduction:**

Huperzine A (HupA) and huperzine B (HupB) are high-value Lycopodiaceae alkaloids with potent, reversible acetylcholinesterase (AChE) inhibitory activity and NMDA receptor antagonism. Their supply is limited by impractical field cultivation and conservation constraints on wild harvesting. Here, we evaluated axenic gametophyte cultures of *Huperzia selago* as a controllable and ethically sourced *in vitro* system and tested LED spectral control as a tool to modulate alkaloid accumulation.

**Methods:**

Cultures were maintained for 24 weeks under three light treatments (WHITE-L, BLUE-L and RED–BLUE-L) or darkness. HupA and HupB were quantified by HPLC–DAD, while LC–MS/IT–TOF and MS/MS-supported profiling were used for target-alkaloid confirmation and characterization of the multi-component extract composition. Extracts standardized to HupA equivalents were evaluated for AChE inhibition.

**Results:**

LED spectral control shaped HupA/HupB accumulation patterns. BLUE-L (≈450 nm) was associated with the strongest increase in HupA content, reaching 4.83 ± 0.21 mg·g^-^¹ DW at week 12, whereas RED–BLUE-L (660/450 nm) maintained the highest mean HupA content over 24 weeks (3.61 ± 0.61 mg·g^-^¹ DW). Darkness promoted relative biomass accumulation, whereas BLUE-L and RED–BLUE-L favored higher HupA/HupB accumulation during the 8–12-week harvest window. These complementary responses suggest a potential two-stage strategy involving biomass accumulation in darkness followed by induction under blue or red–blue light; however, this sequential approach requires future validation. HupB remained a minor but light-responsive metabolite (≤0.74 ± 0.03 mg·g^-^¹ DW). Extracts standardized to HupA equivalents (0.5–2.0 µM) inhibited AChE up to approximately 50%, but HupA content alone did not fully explain the observed activity.

**Discussion:**

The weak-to-moderate relationship between HupA content and AChE inhibition suggests that optimization of *H. selago* gametophyte cultures should consider not only HupA accumulation, but also the broader alkaloid profile and extract bioactivity. LED spectral control supports the development of reproducible, ethically sourced systems for obtaining Lycopodiaceae alkaloid-rich biomass under controlled conditions.

## 1 Introduction

Huperzine A (HupA, selagine) and huperzine B (HupB) are unique alkaloids found exclusively in only a few club mosses (Lycopodiaceae) of the genus *Huperzia* Bernh. and *Phlegmariurus* Holub (Lycopodiaceae P.Beauv. ex Mirb.) (Ma et al., 2005, 2007; Szypuła and Pietrosiuk, 2021a, b). Both alkaloids are potent acetylcholinesterase (AChE) inhibitors and antagonists of the *N*-methyl-D-aspartate (NMDA) receptor and their procognitive potential has been confirmed by numerous clinical studies in patients with Alzheimer’s disease (AD) (Li et al., 2008; Ferreira et al., 2016; Yang et al., 2013; Blanco-Silvente et al., 2017). Most drugs currently used for the symptomatic treatment of AD act by reversible AChE inhibition and/or NMDA receptor antagonism (Ferreira et al., 2016; Majidazar et al., 2022), whereas newer approaches such as monoclonal antibody therapies target other aspects of disease pathology (Van Dyck et al., 2023). AChE inhibition is a clinically accepted strategy enhancing cholinergic transmission with confirmed effectiveness in improving AD symptoms combined with an acceptable safety profile (Miziak et al., 2021; Majidazar et al., 2022). Three AChE inhibitors, donepezil, rivastigmine and galantamine, are used for the symptomatic treatment of mild to moderate AD, but they have some pharmacokinetic limitations (Farlow, 2002; Nordberg et al., 2013). Despite advances in treatment, there remains a need for new potent CNS-penetrant AChE inhibitors, with submicromolar or low micromolar IC_50_ values and favorable pharmacokinetic profiles (Colović et al., 2013; Sharma, 2019). The target alkaloids investigated in this study, (−)-huperzine A (HupA) and (−)-huperzine B (HupB), belong to the most potent known AChE inhibitors, which makes them promising candidates for pharmacotherapy of cognitive impairments.

*Huperzia serrata* (Thunb.) Trevis. remains the main natural source of HupA for the pharmaceutical industry (Ma et al., 2005, 2007). The species is extremely slow to grow, entirely dependent on mycorrhizal associations and the wild populations are small and susceptible to overexploitation (Ma et al., 2005, 2007; Ferreira et al., 2016). The HupA content in dry plant is low (usually in the hundredths of a percent range), and field cultivation of *H. serrata* and other club moss species is obviously impractical. Consequently, sustainable sourcing of HupA/HupB from the wild is impossible to maintain and *in vitro* systems such as micropropagation, callus cultures, cell suspensions and especially gametophyte cultures are the only realistic options.

*Huperzia selago* (L.) Bernh. ex Schrank et Mart., another club moss species, is the primary biotechnological model with established strategies to obtain axenic gametophyte cultures from spores (**Figure 1**) which ensures significant acceleration of the developmental cycle now lasting months, not years. Spore collection causes no damage to the wild populations and gametophyte cultures unlike sporophyte cultures are easier to maintain in axenic conditions (Szypuła et al., 2020; Szypuła and Pietrosiuk, 2021a). Importantly, *H. selago* gametophytes have been shown to accumulate HupA and HupB at levels comparable to or even higher than those reported for many sporophyte-based culture systems, while maintaining sustained biomass accumulation over time. These features make *H. selago* gametophytes a valuable *in vitro* system for obtaining alkaloid-rich biomass under controlled conditions (Szypuła et al., 2020; Szypuła and Pietrosiuk, 2021a).

**Figure 1:**
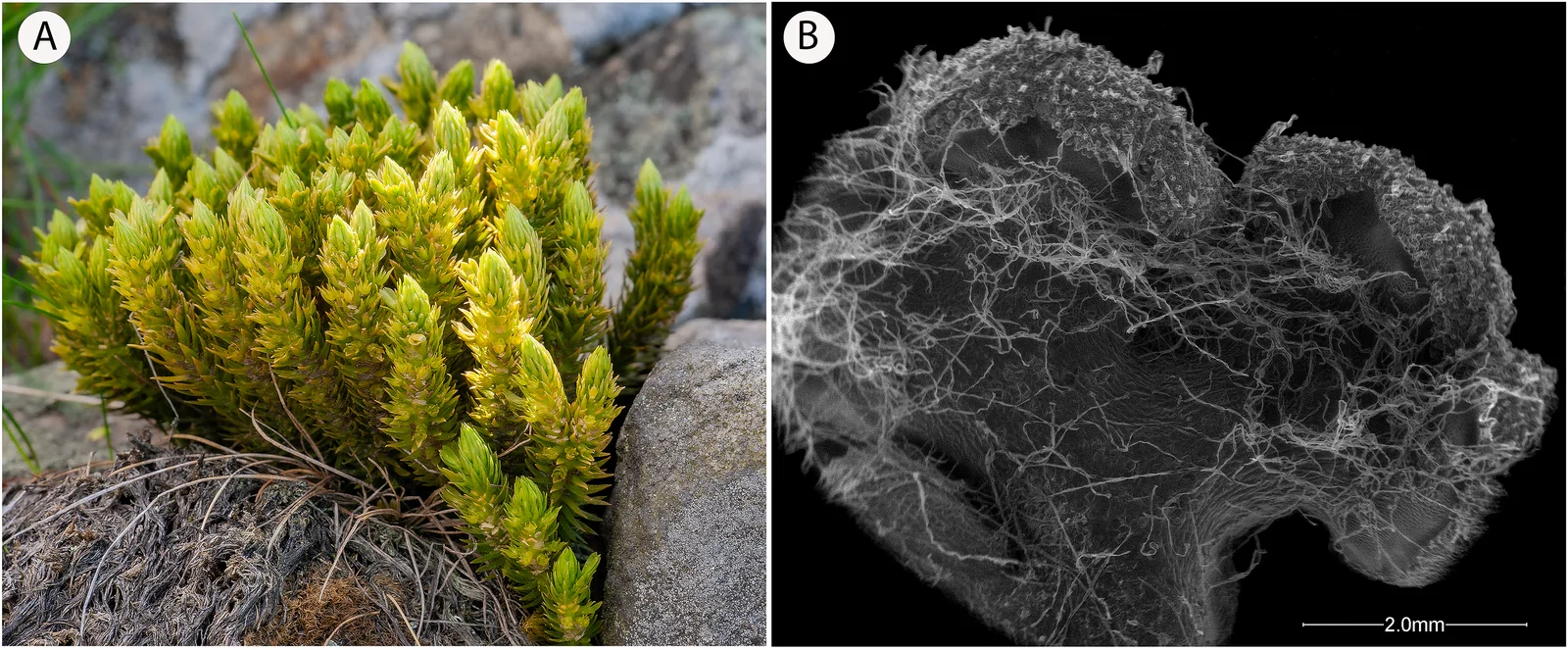
Two autonomous generations of *Huperzia selago*: **(A)** sporophyte (2n) in the spore-producing phase, photographed in its natural habitat; **(B)** axenic gametophyte (1n) obtained *in vitro* from spores (12-week culture on Whittier and Storchová (2007) medium), imaged with a scanning electron microscope (ESEM; Quanta 200 FEI, Eindhoven, Netherlands) on unfixed, hydrated material, enabling high-resolution visualization of surface morphology under near-natural conditions (Photograph: W. Szypuła).

Over the last decade light-emitting diode (LED) illumination has been recognized as one of the most cost-effective tools to control secondary metabolism in *in vitro* cultures (Siatkowska et al., 2021; Peng et al., 2024). The spectral quality of light (the balance of blue and red components) acts on the photoreceptors (cryptochromes, phytochromes and indirectly on phototropin activity), and via signal cascades on the expression of genes associated with nitrogen metabolism, oxidative stress response and biosynthesis of nitrogen compounds, including alkaloids (Siatkowska et al., 2021; Peng et al., 2024; Szypuła and Pietrosiuk, 2025). Blue light is associated with signaling pressure on secondary metabolic pathways, resulting in a shift from biomass growth toward secondary metabolite accumulation, as the synthesis and accumulation of secondary metabolites are prioritized over growth. Red light on the other hand activates the phytochrome system thus affecting the source-outlet relations. It modulates the production of assimilates (source), their loading into the phloem and redistribution to the growth and storage zones (outlet), which translates directly to carbon allocation and balance *in vivo* and *in vitro* (Siatkowska et al., 2021; Rai et al., 2024; Szypuła and Pietrosiuk, 2025). In controlled *in vitro* systems, spectral control allows separating the phase of rapid biomass growth from the induction phase of metabolite accumulation and introducing two-stage culture protocols compatible with photobioreactors (Ramirez-Estrada et al., 2016; Peng et al., 2024). The routine use of LED lighting, especially red and blue LEDs, promotes secondary metabolite accumulation and biomass growth, as confirmed for a number of pharmaceutically important plants (e.g., *Salvia miltiorrhiza*, *Hypericum perforatum*) (Zhang et al., 2020; Karimi et al., 2022; Li et al., 2023).

Despite numerous studies on the effect of light on secondary metabolism in plant *in vitro* cultures, the biotechnological use of club mosses remains much less developed than that of seed plants and many other spore plants. *In vitro* production of cholinesterase-inhibiting compounds in Lycopodiaceae is still a relatively new and rarely explored research area (Szypuła and Pietrosiuk, 2021a), and gametophyte-based systems have been investigated only to a limited extent (Freeberg and Wetmore, 1957; Whittier and Storchová, 2007; Szypuła et al., 2020; Szypuła, 2023). This is particularly important because Lycopodiaceae gametophytes are biologically unusual haploid structures, often subterranean, slow-developing and fungus-dependent under natural conditions. Therefore, axenic gametophyte cultures of *Huperzia selago* provide a unique model for studying HupA/HupB accumulation under controlled trophic and environmental conditions. To the best of our knowledge, defined LED spectral regimes and darkness have not previously been evaluated as modulators of HupA/HupB accumulation, relative biomass growth, multi-component alkaloid profiles and AChE-inhibitory activity in this gametophyte-based system.

The current study employed axenic cultures of *Huperzia selago* gametophytes as an *in vitro* system for studying HupA/HupB accumulation, and investigated the effects of three light treatments (white light, λ = 450/550/660 nm; blue light, λ = 450 nm; red-blue light, λ = 660/450 nm) and darkness on the kinetics of HupA/HupB accumulation over time (as determined by HPLC-DAD with LC–MS/IT–TOF confirmation), biomass growth, and anticholinesterase-inhibitory activity of the standardized extracts (the Ellman assay). We also analyzed the linear relationship between the HupA content and the percentage of AChE inhibition using Pearson and Spearman correlations, and linear modeling.

Considering potential applications, the overall aim was to evaluate whether continuous light treatments and darkness can be used to identify culture conditions associated with biomass accumulation, HupA/HupB accumulation and AChE-inhibitory activity of standardized extracts. On this basis, we aimed to indicate a potential harvest window and to define parameters that may support the future experimental testing of a two-stage strategy, involving biomass accumulation in darkness followed by metabolite induction under blue or red–blue light. Such a sequential strategy was not tested directly in the present study and should therefore be treated as a hypothesis for further optimization, including possible future transfer to photobioreactor systems.

Summing up, the present experimental approach combines controlled photomodulation, alkaloid profiling and bioactive functional testing to investigate the regulation of HupA/HupB accumulation in *Huperzia selago* gametophytes and to provide a basis for future optimization of controlled *in vitro* strategies for obtaining bioactive Lycopodiaceae alkaloid-rich biomass.

## 2 Materials and methods

### 2.1 Reagents and standards

The following reagents and reference standards were used in this study: acetylcholinesterase (AChE) (Sigma-Aldrich Chemie GmbH, Steinheim, Germany); acetylthiocholine iodide (ATChI) (Sigma-Aldrich Chemie GmbH, Steinheim, Germany); 5,5′-dithiobis(2-nitrobenzoic acid) (DTNB) (Merck KGaA, Darmstadt, Germany); physostigmine salicylate (Sigma-Aldrich Chemie GmbH, Steinheim, Germany); dimethyl sulfoxide (DMSO) (Sigma-Aldrich, Germany); Plant Preservative Mixture (PPM™) (Plant Cell Technology, Washington, DC, USA); Tris-HCl (Tris(hydroxymethyl)aminomethane hydrochloride) and D-(+)-glucose (both from Merck KGaA, Darmstadt, Germany); methanol, HPLC grade (Chromasolv^®^) (Merck KGaA, Darmstadt, Germany); sodium hexafluorophosphate (NaPF_6_) (Merck KGaA, Darmstadt, Germany); ammonium acetate (Merck KGaA, Darmstadt, Germany); water, HPLC grade (Merck KGaA, Darmstadt, Germany); water, LC–MS grade (18.2 MΩ·cm; Milli-Q^®^, Millipore Merck KGaA, Darmstadt, Germany); acetonitrile, LC–MS grade (Merck KGaA, Darmstadt, Germany); Bacto™ Agar (Becton, Dickinson and Company, Franklin Lakes, NJ, USA). Huperzine A and Huperzine B analytical standards were purchased from ChromaDex, Inc. (Laguna Hills, CA, USA). Unless otherwise stated, reagents were of analytical grade; chromatographic solvents were HPLC or LC–MS grade as indicated.

### 2.2 Plant material and establishment of *in vitro* culture

The *in vitro* cultures of *H. selago* gametophytes (prothalli) were initiated in earlier studies using spores (Szypuła et al., 2020). In all experiments the gametophytes were cultured on the solid basal medium according to Whittier and Storchová (2007) with the addition of 5 g L^-1^ glucose. The pH of the medium was adjusted to 5.9 prior to autoclaving (121 °C under 1 bar overpressure (15 psi) for 15 minutes) and the medium was solidified with agar (1.1%; w/v). To prevent microbial contamination in the course of long-running experiments 2 ml·L^-1^ of Plant Preservative Mixture™ (PPM™; Plant Cell Technology, Washington, USA), a broad-spectrum biocide, was added to the media. The cultures were incubated in growth chambers at 18 ± 1 °C (day) and 16 ± 1 °C (night) with a 16-hour photoperiod (50 μmol·m^-2^·s^-1^) or without light. The gametophytes were transferred on to fresh media of the same composition every 2 months, and the duration of each experiment was 24 weeks (6 months). At weeks 0, 4, 8, 12, and 24, gametophyte fresh weight (FW) was expressed as a relative fresh-weight index (RFW, %) based on 30 gametophytes per condition and sampling time (n = 30). The experiment was performed in three independent biological replicates (N = 3). RFW (%) was calculated as: RFW (%) = (FWt/FW0) × 100, where FW0 is the initial fresh weight (week 0) and FWt is the fresh weight at time t. Values are reported as mean ± SD (N = 3).

### 2.3 Light sources

Light treatments were conducted in growth chambers with adjustable Photosynthetic Photon Flux Density (PPFD) using customized LED panels (RGB + white) equipped with Total Internal Reflection (TIR) collimators (≈20–25 mm diameter; 24 emitters per 40 × 30 cm panel) mounted 20 cm above the culture vessels. Panels were powered by MEAN WELL LPF-60D-48 (48 V, 1.25 A, IP67, 3-in-1 dimming) and MEAN WELL LPF-25D-24 (24 V, 1.05 A, IP67, 3-in-1 dimming) constant-voltage drivers. Three spectral variants were tested: 1) WHITE-L – mixed spectrum composed of 40% white LEDs (CCT 6400 K), 20% red (λ = 660 nm), 20% green (λ = 550 nm), and 20% blue (λ = 450 nm); 2) RED–BLUE-L – red-blue lighting with 50% red (λ = 660 nm) and 50% blue (λ = 450 nm) and 3) BLUE-L – blue-only lighting (100%, λ = 450 nm). The selected spectral regimes were chosen to compare a broad white-light reference treatment with blue-light exposure and a red–blue combination. BLUE-L was included because blue light is frequently associated with the stimulation of secondary-metabolite accumulation, whereas RED–BLUE-L represents one of the most commonly used LED combinations in controlled plant *in vitro* culture and combines blue-light signaling with a red-light component relevant to photosynthetic and phytochrome-mediated responses. Monochromatic red light, far-red light and other spectral combinations were not included in this initial 24-week time-course experiment in order to keep the experimental design focused and statistically manageable.

WHITE-L was used as the white-light reference treatment, whereas cultures maintained without illumination were designated as the dark treatment (DARK). Spectral characteristics and irradiance at the culture plane were verified with an Apogee Instruments QMSWSS quantum meter model at 20 cm distance; Photosynthetic Photon Flux Density (PPFD) was 50 µmol m^-^²s^-^¹ for each treatment.

### 2.4 Alkaloid extraction and determination

#### 2.4.1 Plant material

Plant material for phytochemical analysis was collected every two weeks during the 24 weeks of culture maintenance, from each batch of gametophytes cultured under different light treatments and from the dark treatment. Until further assays, the collected gametophytes were kept in an ultralow temperature freezer at -80°C.

#### 2.4.2 Preparation of extracts and their purification

The alkaloids were extracted from the plant material using the modified methods we described earlier (Szypuła et al., 2011, 2020). In this modification, the lyophilized and powdered plant material (0.25 g on average) was transferred to glass test tubes and extracted three times with HPLC grade methanol, added in decreasing amounts (5 mL, then 4 mL, then 3 mL) during successive repetitions. At each repetition, the material was sonicated in a Sonorex ultrasonic bath at 40°C. Each extraction was performed for 30 minutes. After each extraction, the methanol extracts were pipetted out and evaporated under reduced pressure. The dry extract was dissolved in a small amount of HPLC grade methanol to a final concentration of approximately 1 mg·mL^- 1^.

#### 2.4.3 Qualitative and quantitative HPLC-DAD analyses of HupA and HupB

Qualitative and quantitative HPLC–DAD analyses of HupA and HupB were performed on a Shimadzu system consisting of a UV-VIS and SPD-10A DAD 340S detector, LC-10AD pump and LabSolution software. A Hypersil GOLD column, C_18_ 250×4.6 mm, and a Hypersil GOLD precolumn (10 × 4 mm, 5 µm), were used. The mobile phase consisted of (A) water with the addition of 30 mM sodium hexafluorophosphate (NaPF_6_) and (B) acetonitrile. The following gradient was applied: 0–5 min 0→25% B, 5–20 min 25→45% B, 20–30 min 45→80% B (Szypuła et al., 2011). Column temperature: 24 ± 1 °C, flow rate of the mobile phase: 1.0 mL·min^–1^, analysis duration: 30 min, detection at wavelengths λ = 210, 230, 260 and 308 nm. The mean HupA content in the gametophytes used to initiate subcultures in this experiment was 2.20 ± 0.11 mg·g^-^¹ DW (Szypuła et al., 2020). For HPLC–DAD quantification, plant material from three independent culture experiments was treated as biological replicates, whereas repeated chromatographic measurements of the same extract were treated as technical replicates and averaged before statistical analysis. Representative HPLC–DAD chromatograms of authentic HupA and HupB standards and a representative *H. selago* gametophyte extract are provided in **Supplementary Figure S1**.

#### 2.4.4 LC–MS/IT–TOF analysis and MS/MS fragmentation of alkaloids

LC–MS/IT–TOF analysis was performed using a Shimadzu Prominence high-performance liquid chromatograph coupled with an LC–MS/IT–TOF mass spectrometer equipped with an electrospray ionization source, an ion trap and a time-of-flight detector. Mass spectra were recorded in the positive-ion mode using LCMSsolution software.

Chromatographic separation was performed on a Kinetex C18 column, 2.6 µm, 2.1 × 100 mm (Phenomenex). The injection volume was 5 or 10 µL, the column oven temperature was maintained at 40 °C, and the flow rate was 0.15 mL·min^-^¹. PDA detection was recorded in the range of 200–800 nm. The mobile phase consisted of water with the addition of 5 mM ammonium formate, pH 4.0 (A), and acetonitrile (B). The following gradient was applied: 0–1 min, 0→3% B; 3–40 min, 3→30% B; 40–50 min, 30→95% B; 50–55 min, 95% B; 55–57 min, 95→3% B, followed by 13 min equilibration at 3% B. The total analysis time was 70 min.

The LC–MS/IT–TOF conditions were as follows: mass range, m/z 100–800; accumulation time, 10 ms; interface temperature, 220 °C; heat block temperature, 220 °C; nebulizing gas flow, 1.5 L·min^-^¹; drying gas pressure, 100 kPa; ion-spray voltage, +4.5 kV. The LC–MS/IT–TOF mass spectrometer was calibrated using the calibration mixture recommended for the system. Before analysis, samples were prepared in acetonitrile:5 mM ammonium formate, pH 4.0, 1:1 (v/v), vortexed and centrifuged, and the supernatants were transferred to LC vials.

For qualitative alkaloid profiling, protonated molecular ions [M+H]^+^ were used for accurate-mass measurement and molecular-formula prediction. MS/MS fragmentation was acquired in the positive-ion mode for selected precursor ions when the signal intensity was sufficient to obtain a reliable tandem mass spectrum. In low-intensity features, for which no reliable MS/MS spectrum could be acquired, annotation was based on accurate-mass measurement, molecular-formula assignment and comparison with literature data.

Huperzine A and huperzine B were confirmed by comparison with authentic standards in HPLC–DAD analysis and additionally supported by accurate mass and MS/MS fragmentation. Other Lycopodiaceae alkaloids were annotated putatively on the basis of accurate mass, predicted molecular formula, MS/MS fragmentation where available, and comparison with previously reported Lycopodiaceae alkaloids. Identification confidence was assigned according to MSI criteria: Level 1 for compounds confirmed with authentic standards, Level 2 for putatively identified compounds or isomer sets supported by characteristic MS/MS fragmentation, and Level 3 for putative annotations based mainly on accurate-mass/formula match, with or without non-diagnostic MS/MS data.

#### 2.4.5 Enzymatic assays of acetylcholinesterase inhibition

Acetylcholinesterase (AChE) activity was determined colorimetrically following Ellman et al. (1961) using a 96-well microplate reader (BioTek Synergy 4). Hydrolysis of acetylthiocholine iodide (ATChI) by AChE generates thiocholine, whose thiol group reacts with 5,5′-dithiobis(2-nitrobenzoic acid) (DTNB) to yield the yellow 5-thio-2-nitrobenzoate (TNB) anion; absorbance was monitored at 412 nm. Tested samples were crude extracts standardized to huperzine A (HupA) equivalents at 0.5, 1.0, 1.5 and 2.0 µM; pure HupA at the same concentrations served as the reference inhibitor. For the AChE assay, extracts prepared independently from three culture experiments were used as biological replicate extracts for each light treatment and sampling week. Each biological replicate extract was adjusted to four HupA-equivalent concentrations: 0.5, 1.0, 1.5 and 2.0 µM. Each concentration of each biological replicate extract was measured in 10 technical wells. Percentage inhibition was calculated from initial reaction rates. Technical well readings were averaged within each biological replicate extract and concentration before statistical analysis. Individual well readings were treated only as technical replicates and were not considered independent biological observations. Unless stated otherwise, AChE inhibition values are reported as mean ± SD of three biological replicate extracts for each light treatment × sampling week × extract concentration combination.

Tris-HCl buffer (0.05 M, pH 7.8) was freshly prepared and stored for up to 48 h at 4 °C. DTNB (7.1 mg) was dissolved in 30 mL of this buffer (≈0.6 mM), and ATChI (8.7 mg) was dissolved in 10 mL of buffer (3 mM). AChE from *Electrophorus electricus* (Type V-S; 744 U mg^-^¹ lyophilized powder; Sigma-Aldrich) was diluted in buffer to 0.30 U·mL^-^¹ and kept on ice (≈4 °C) during use. The HupA stock was prepared by dissolving 1.00 mg in 50 µL DMSO and bringing to 1.00 mL with buffer (1.0 mg mL^-^¹), then serially diluting to 0.5–2.0 µM. Into each well were added, in order: 125 µL DTNB (0.6 mM), 50 µL Tris-HCl buffer (0.05 M), 25 µL extract or HupA standard, and 25 µL AChE (0.30 U·mL^-^¹). After 5 min pre-incubation at 37 °C, the reaction was started by adding 25 µL ATChI (3 mM). Absorbance at 412 nm was recorded every 30 s for 5 min, and slopes (ΔA/min) were used to compute % inhibition relative to the vehicle control. Vehicle controls contained buffer with DMSO at the highest assay concentration (≤0.5% v/v).

To control for matrix interference and correct the signal, for each sample, the following controls were run in parallel: (1) enzyme-free wells (substrate + DTNB ± extract) to correct for extract self-absorbance at 412 nm and spontaneous ATChI hydrolysis; (2) substrate-free blanks (DTNB ± extract + enzyme) to detect any direct DTNB reduction; and (3) solvent controls (buffer + DMSO). Reaction rates were corrected by subtracting the corresponding control slopes before calculating % inhibition.

### 2.5 Statistics

All analyses were performed in TIBCO^®^ Statistica v13/v14 (TIBCO Software Inc., Palo Alto, CA, USA). Data are reported as mean ± SD. For biomass accumulation and HupA/HupB content, each experimental condition was evaluated in three independent experiments, with technical replicates averaged to the experiment level before statistical testing. Between light treatments within a given week and across weeks within a treatment, one-way ANOVA was used; in the case of variance heterogeneity, Welch’s ANOVA was applied. If normality or homoscedasticity assumptions were violated, the Kruskal–Wallis test followed by Dunn’s *post hoc* test with Bonferroni correction was used. *Post hoc* comparisons were performed using Tukey’s HSD test for all-pair comparisons or Dunnett’s test versus the white-light reference treatment, as specified in figure and table captions.

For AChE inhibition assays, extracts prepared independently from three culture experiments were treated as biological replicate extracts for each light treatment and sampling week. Technical well readings were first averaged within the corresponding biological replicate extract and concentration. Thus, individual well readings were treated only as technical replicates and were not used as independent observations in statistical testing. For the treatment-level comparison reported in **Table 1**, replicate-level values were summarized at the light treatment × sampling week × concentration level, and comparisons among light treatments were performed on week × concentration means using the Kruskal–Wallis test. The relationship between AChE inhibition (%) and HupA content (mg·g^-^¹ dry extract) was assessed by Pearson and Spearman correlations and ordinary least squares (OLS) linear regression (% AChE inhibition = β_0_ + β_1_·HupA), reporting r or ρ, R² and p values. Statistical significance was set at two-sided p < 0.05.

**Table 1:** Mean acetylcholinesterase (AChE) inhibition (%) and mean HupA content (mg·g^-^¹ dry extract) in *Huperzia selago* gametophyte extracts.

Light condition	AChE inhibition [%]	HupA [mg·g^-^¹ dry extract]
WHITE-L	19.88 ± 12.75	13.00 ± 2.15
RED–BLUE-L	21.69 ± 11.58	13.75 ± 1.44
BLUE-L	21.58 ± 8.72	13.77 ± 1.52
DARK	23.13 ± 12.51	14.26 ± 2.33

For the AChE assay, three independently prepared biological replicate extracts were used for each light treatment and sampling week, and each extract was tested at four HupA-equivalent concentrations. Technical well readings were first averaged within each biological replicate extract and concentration; biological replicate means were then averaged to obtain one value for each light treatment × sampling week × extract concentration combination. AChE values in the table are mean ± SD calculated from these week × concentration means for each light treatment (n = 48; 12 sampling weeks × 4 extract concentrations). Differences among light treatments were assessed using the Kruskal–Wallis test. No statistically significant differences were detected among light treatments (H(3, N = 192) = 1.87, p = 0.6006).

Color shading in the first column corresponds to the color coding of the light treatments used in the figures and has no statistical meaning.

## 3 Results

### 3.1 *In vitro* cultures

The subcultures of *H. selago* gametophytes presented high viability as with each transfer 90–100% of the explants survived and continued to develop without any signs of necrosis or contamination while mature gametophytes demonstrated evident polymorphism. Most frequently disk-shaped or cylindrical structures were observed with distinct dorsal and ventral surfaces, and an hour-glass cross-section, although there were also some heart-, carrot- and pincushion-shaped gametophytes (**Figure 2**). In most of the prothalli the ventral surface was densely covered with trichomes but in some rare cases the ventral surface was devoid of trichomes, which additionally confirms the phenotypic differences between cultures.

**Figure 2:**
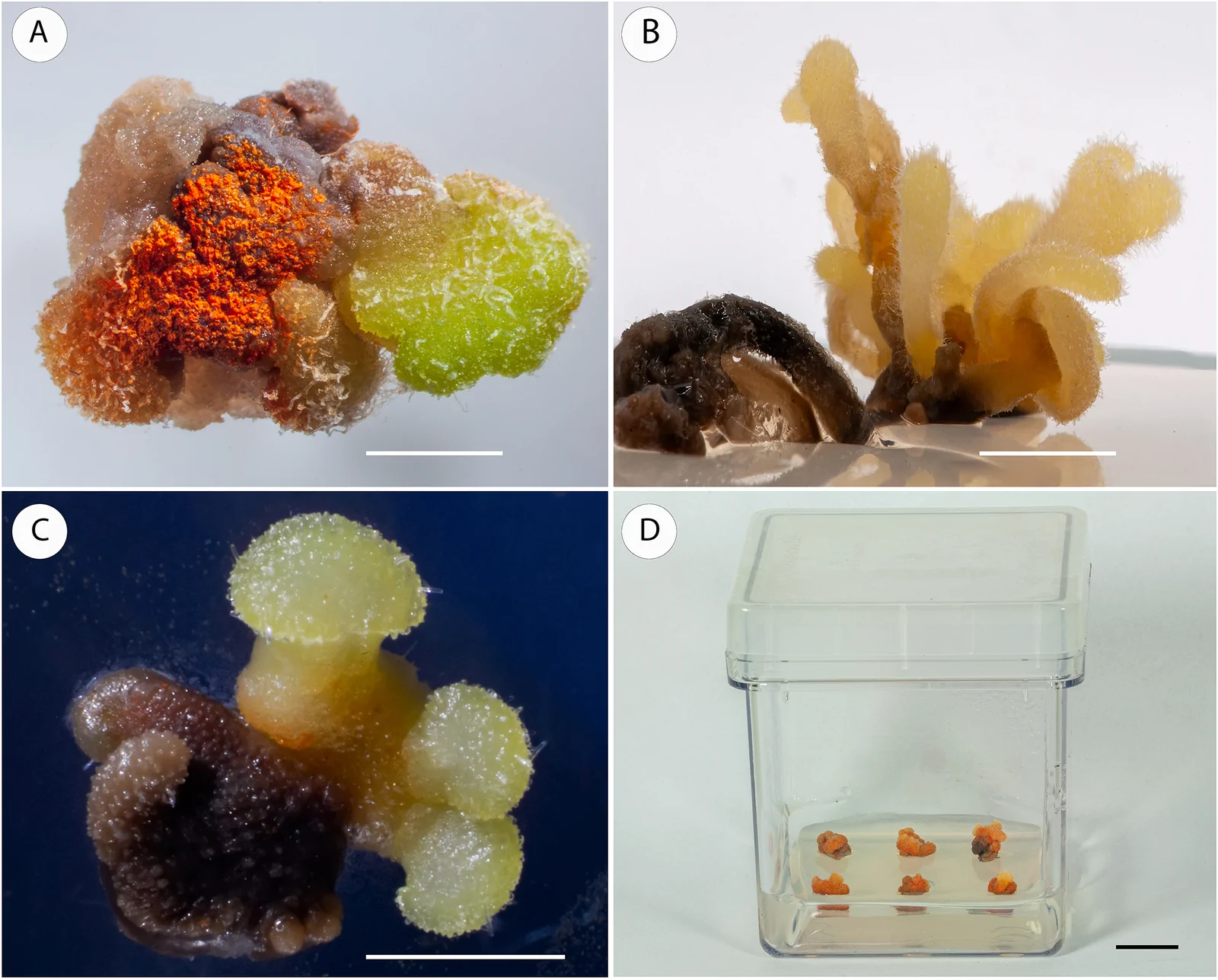
Morphological polymorphism of *H. selago* gametophytes in *in vitro* culture. **(A)** disk-shaped forms; orange colored crystalline alkaloid secretion produced by gametophyte tissue seen on the surface. **(B)** Proliferating cylindrical forms with clearly visible dorsal and ventral surfaces. **(C)** Heart-shaped form with a wavy edge, numerous paraphyses and gametangia seen on the surface. **(D)** Container (Ø 7 cm) with four-week-old prothalli from the same subculture on medium according to Whittier and Storchová (2007) in a LED growth chamber. Scale: 2 mm **(A–C)** and 1 cm **(D)**.

Throughout the 24-week experiment, all light treatments and darkness promoted biomass accumulation in *H. selago* gametophytes, but their effectiveness differed depending on the wavelength (**Figure 3**). As early as 4 weeks, the fresh mass increased 1.9–2.2-fold (190–215%) and by week 8, the increase had become stable at a level of 3.5-4.00 times the initial value (week 0).

**Figure 3:**
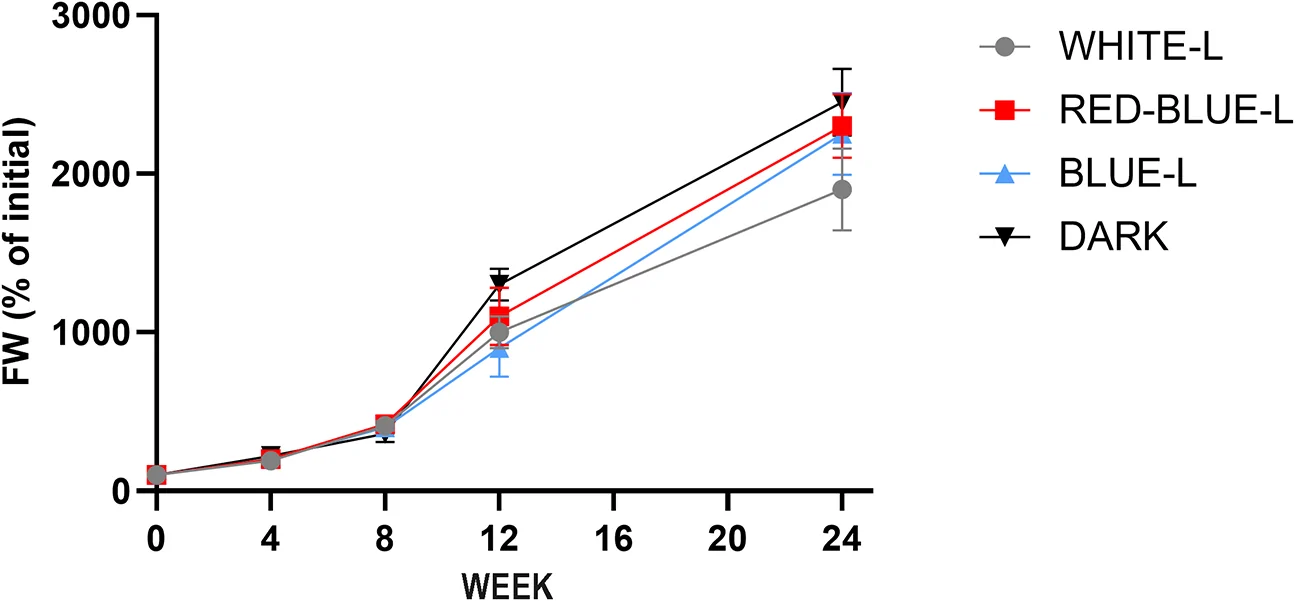
Kinetics of changes in the *H. selago* gametophyte fresh weight during 24 weeks *in vitro* culture. The curves represent the mean growth (%) relative to the initial value (week 0 = 100%) under four experimental regimes: WHITE-L (gray), RED–BLUE-L (red), BLUE-L (blue) and DARK (black).

After 12 weeks the differences in the growth rates could be observed: under DARK the growth was 13.0-fold, outdistancing RED–BLUE-L treatment (11.2-fold growth) and WHITE-L treatment (9.8-fold growth). At the last measurement at 24 weeks, the highest biomass accumulation was again observed in darkness (24.5-fold), while the combination of red and blue light (RED–BLUE-L = ×23.00) and blue light (BLUE-L = ×22.9) were only marginally inferior. The biomass growth was significantly lower (19.0-fold) in gametophytes exposed to white light.

Logarithmic transformation confirms that the key growth rate differentiation occurs between weeks 8 and 12, with the highest relative growth observed under the dark treatment (DARK). The data collected show that darkness or exposure to a light spectrum enhanced with red and/or blue light promoted intense growth of gametophyte fresh mass while a long-term exposure to white light slowed down the process.

### 3.2 Phytochemical assays

Alkaloids isolated from *H. selago* gametophytes obtained *in vitro* under different experimental regimes were investigated using two complementary chromatographic and mass-spectrometric methods. LC–MS/IT–TOF was used for high-resolution qualitative profiling of the alkaloid fraction, accurate-mass measurement, molecular-formula prediction and, where possible, MS/MS-based support of putative annotations. HPLC–DAD was used for qualitative confirmation with authentic standards and quantitative, time-resolved monitoring of HupA and HupB concentrations at different time points of the experiment. These qualitative and quantitative data enabled characterization of the multi-component alkaloid profile, determination of HupA and HupB accumulation kinetics and selection of the light treatment associated with the highest HupA/HupB accumulation response.

#### 3.2.1 Mass-spectrometric characterization (LC–MS/IT–TOF) of the alkaloid profile

The LC–MS/IT–TOF analysis confirmed that the alkaloid fraction of *H. selago* gametophytes obtained *in vitro* was complex and included at least 18 detectable alkaloid features in the positive-ion ESI mode (**Figure 4**; **Supplementary Table S1**). The qualitative profiles of extracts obtained from gametophytes cultured under different light regimes were generally similar, although the relative contribution of individual alkaloids differed between samples. The detected compounds represented the main structural groups of Lycopodiaceae alkaloids, including huperzine/lycodine-type, lycopodine-type, fawcettimine-type and other related alkaloids.

**Figure 4:**
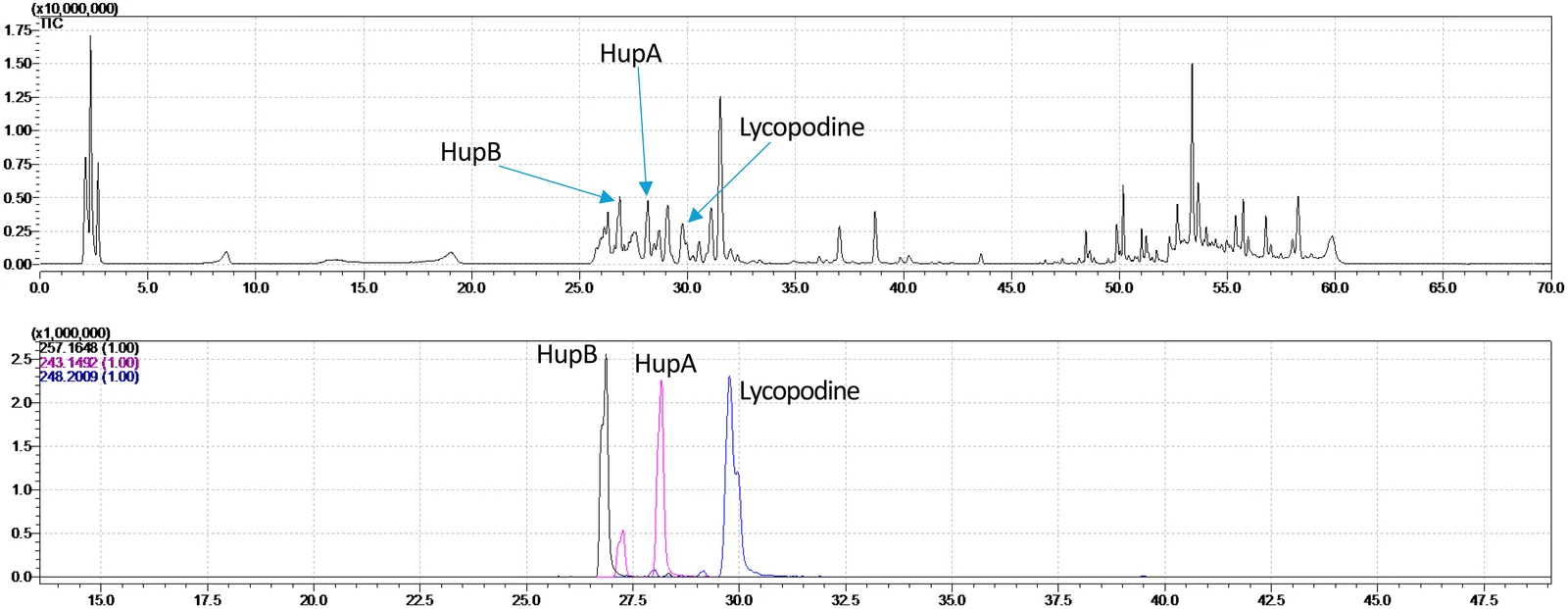
Total ion chromatogram (TIC, ESI^+^) of a representative *Huperzia selago* gametophyte extract; arrows mark peaks assigned to HupB, HupA and a lycopodine/selagoline-type alkaloid. Bottom: extracted-ion chromatograms (EICs) for HupB [M+H]^+^, m/z 257.1647, t*_R_* 26.8 min; HupA [M+H]^+^, m/z 243.1488, t*_R_* 28.1 min; and lycopodine/selagoline-type alkaloid [M+H]^+^, m/z 248.2000, t*_R_* 29.8 min.

Huperzine A and huperzine B were the only compounds confirmed at MSI Level 1. HupA was detected as the protonated molecule [M+H]^+^ at m/z 243.1481–243.1488, whereas HupB was detected as [M+H]^+^ at m/z 257.1642–257.1647. Their identity was confirmed by comparison with authentic standards in HPLC–DAD analysis and additionally supported by accurate-mass measurement and MS/MS fragmentation (**Figure 5**). Representative HPLC–DAD chromatograms documenting the retention behavior of authentic HupA and HupB standards and their detection in a representative gametophyte extract are shown in **Supplementary Figure S1**. The MS/MS spectrum of HupA contained product ions at m/z 227, 210, 196, 192, 178, 165, 154, 115 and 91, whereas the MS/MS spectrum of HupB included product ions at m/z 240, 212, 198, 184, 158, 154, 130, 115 and 91. These data confirmed the presence of the target huperzines in the gametophyte extracts and supported their use as quantitative marker compounds in the time-course HPLC–DAD analysis.

**Figure 5:**
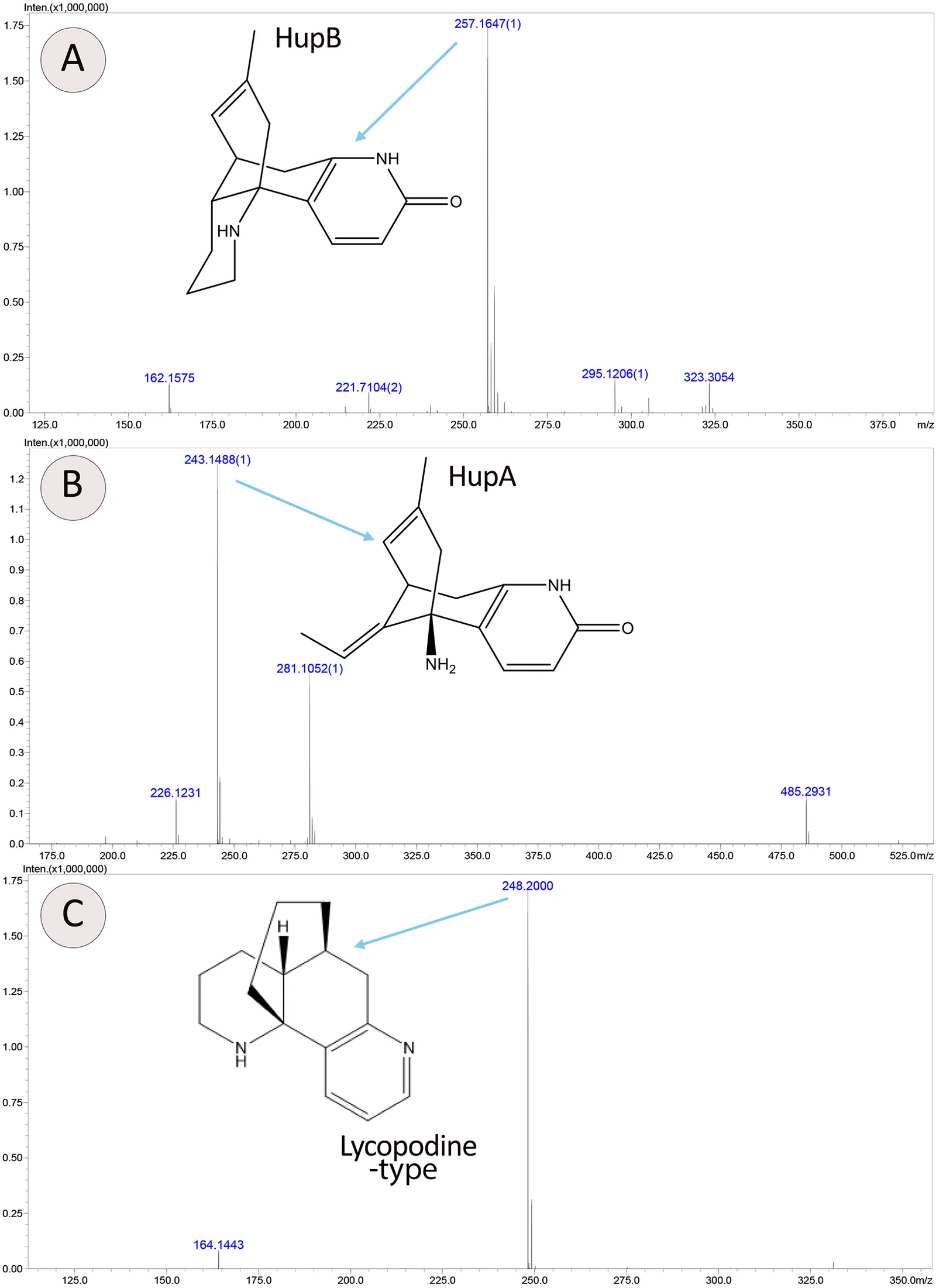
Positive-ion ESI mass spectra of selected alkaloid features detected in a representative *Huperzia selago* gametophyte extract: **(A)** huperzine B, detected as [M+H]^+^ at m/z 257.1647, tR = 26.80 min; **(B)** huperzine A, detected as [M+H]^+^ at m/z 243.1488, tR = 28.13 min; and **(C)** a lycopodine/selagoline-type alkaloid, detected as [M+H]^+^ at m/z 248.2000, tR = 29.79 min.

In addition to HupA and HupB, several co-occurring Lycopodiaceae alkaloids were detected. A lycopodine/selagoline-type feature was detected as [M+H]^+^ at m/z 248.1998–248.2000 and showed MS/MS fragmentation consistent with a lycopodine-type skeleton. Other detected alkaloid features were assigned putatively to fawcettimine-type alkaloids, oxygenated lycopodine-type alkaloids, lycoposerramine-related isomers, lycodine/huperzine-related isomers, flabelline and other Lycopodiaceae alkaloids. For several of these features, MS/MS spectra acquired in the present study provided additional support for the proposed alkaloid class or isomeric candidate set.

However, except for HupA and HupB, most alkaloids could not be assigned at the definitive structure level because several Lycopodiaceae alkaloids share the same elemental composition and may produce partly overlapping fragmentation patterns. Therefore, the non-target alkaloids were reported as putative annotations or isomeric candidate sets. Compounds supported by characteristic MS/MS fragmentation were assigned as MSI Level 2 or Level 3 depending on the diagnostic value of the fragmentation pattern, whereas low-intensity features without reliable MS/MS spectra were retained at MSI Level 3. This conservative annotation strategy avoids overinterpretation of isomeric Lycopodiaceae alkaloids while documenting the multi-component character of the extracts.

The presence of several co-extracted Lycopodiaceae alkaloids is important for the interpretation of the biological activity of the extracts. Although HupA was the main quantified target alkaloid, the observed AChE-inhibitory activity may not be attributable exclusively to HupA, but may also involve HupB and other structurally related alkaloids occurring in the gametophyte extracts.

#### 3.2.2 HupA and HupB content

HPLC-DAD chromatogram peaks corresponding to HupA and HupB were characterized by their specific retention, selectivity and resolution. The mean retention time for the standards was 11.08 ± 0.10 min for HupB and 11.54 ± 0.11 min for HupA. The retention times for the alkaloids detected in the extracts were 11.00 ± 0.15 min for HupB and 11.50 ± 0.20 min for HupA. The UV spectra of both standard HupA and HupB and the corresponding alkaloids detected in the extracts were identical; recorded over 190–800 nm, they displayed absorption maxima at 231.8 nm and 307.7 nm for HupA, and at 222.9 nm and 306.8 nm for HupB.

These results indicate a very high HupA content for the gametophytes obtained *in vitro*, ranging from 1.26 ± 0.01 to 4.83 ± 0.21 mg·g^-1^ DW gametophyte. As shown in **Figure 6**, the highest HupA content, as high as 4.83 ± 0.21 mg·g^-1^ DW, was found in the gametophytes cultured under blue light (BLUE-L) at 12 weeks in culture and the lowest content of 1.26 ± 0.01 mg·g^-1^ DW after 14 weeks under white light (WHITE-L) (upper panel of **Figure 6**).

**Figure 6:**
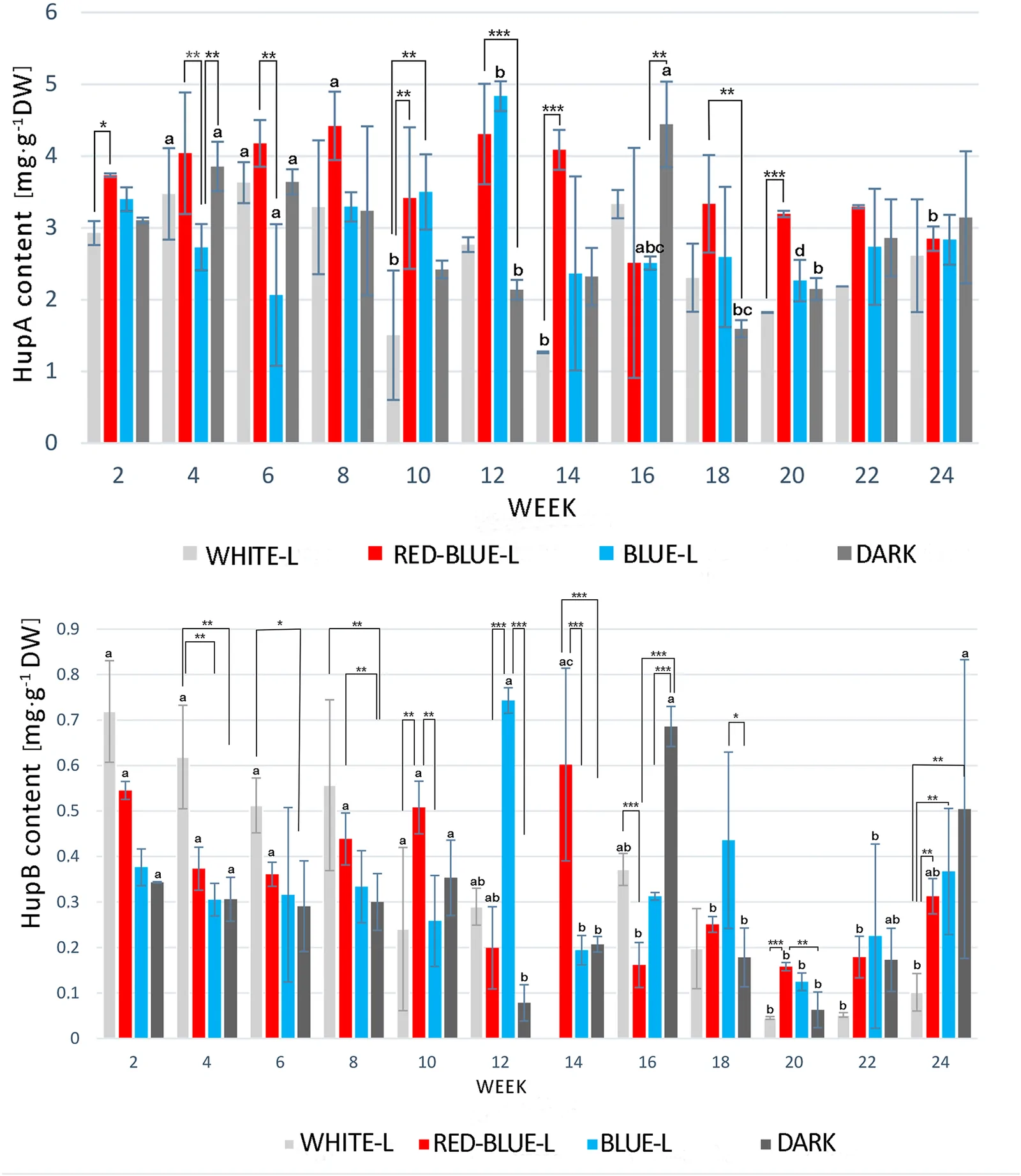
Accumulation of huperzine A (HupA) and huperzine B (HupB) in *Huperzia selago* gametophytes cultured *in vitro* for 24 weeks under four experimental regimes: WHITE-L (white), RED–BLUE-L (red), BLUE-L (blue) and DARK (darkness). Alkaloid content is expressed as mg·g^-^¹ DW. Bars = mean ± SD; n = 3 independent experiments, each with 3 technical replicates. Comparison among experimental regimes within each week was performed using one-way ANOVA followed by Tukey’s HSD; brackets indicate significant differences. Comparison between weeks within a given light regime: one-way ANOVA (factor: week), Tukey HSD; the same letters over the bars indicate homogeneous groups (no significant differences); different letters indicate statistically significant differences (*p* < 0.05). Bars without letters indicate inclusion in a given homogeneous group (ns). Asterisks indicate significance levels: one asterisk (*) indicates p < 0.05, two asterisks (**) indicate p < 0.01, and three asterisks (***) indicate p < 0.001.

The highest mean HupA accumulation was observed in cultures under a mixed light treatment (RED–BLUE-L), as much as 3.61 ± 0.61 mg·g^-1^ DW gametophyte (p < 0.001), and the lowest mean value was observed under the white-light reference treatment (WHITE-L) (2.59 ± 0.79 mg·g^-1^ DW) (**Figure 7**). The mean HupA contents in gametophytes cultured under blue light (BLUE-L) and in darkness (DARK) were comparable, reaching 2.93 ± 0.75 and 2.91 ± 0.82 mg·g^-^¹ DW, respectively.

**Figure 7:**
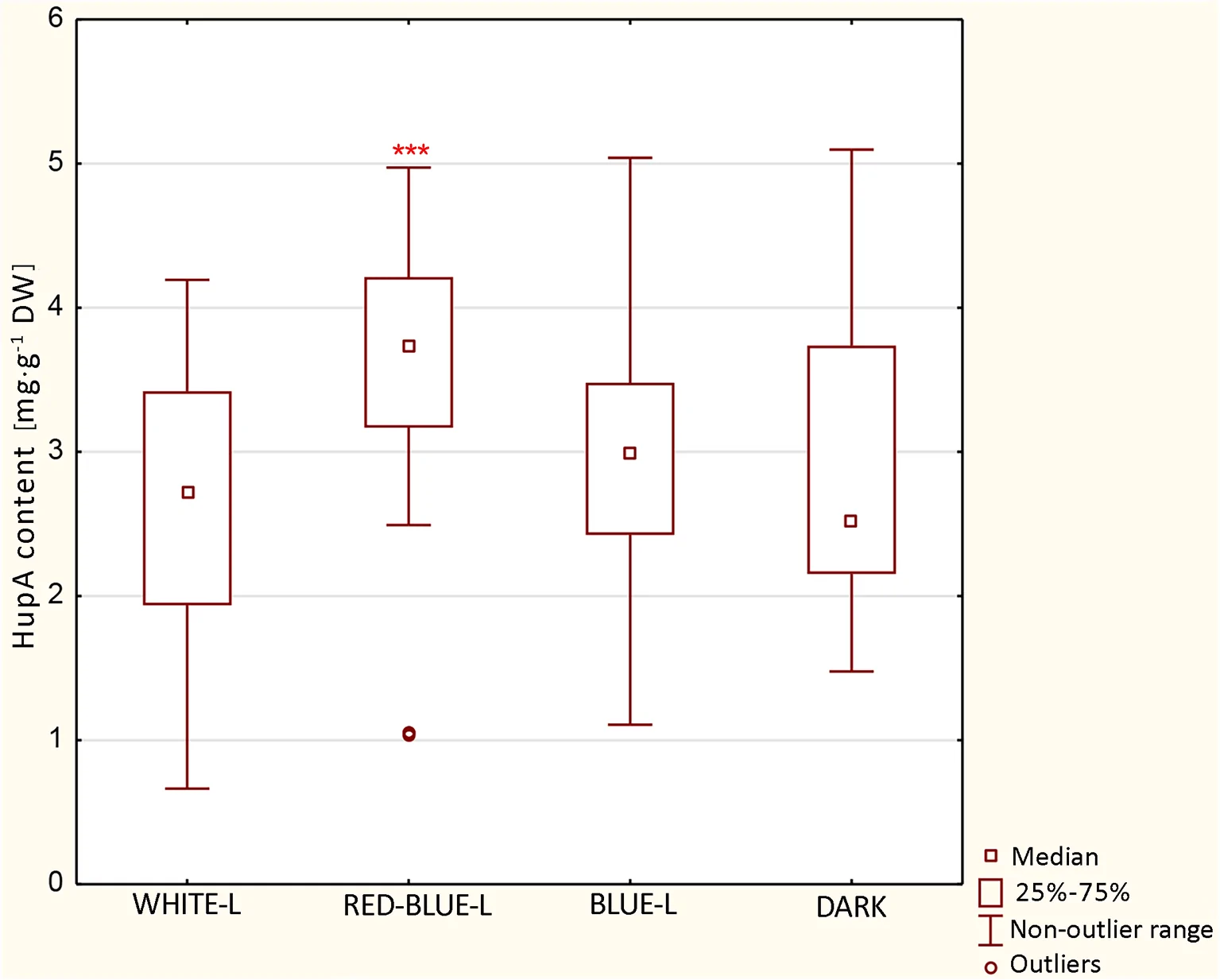
Distribution of huperzine A content (mg·g^-^¹ DW) in *Huperzia selago* gametophytes cultured *in vitro* under four experimental regimes. The box-and-whisker plots depict the median (□), interquartile range (25–75%), non-outlier range (whiskers) and outliers (•). The RED–BLUE-L treatment was associated with significantly higher HupA content than the white-light reference treatment (WHITE-L) (***), one-way ANOVA (experiment-level means) followed by Dunnett’s test; ***p < 0.001. n = 3 independent experiments (the mean of 3 replicates per experiment).

A general trend of changes in the HupA content over 24 weeks of *H. selago* gametophyte culture is depicted in **Figure 8**. Although there were considerable differences in the increases in the HupA content between the light treatments and the experiment weeks, a downward trend was noted in the alkaloid levels in consecutive weeks. Statistical analyses showed that, apart from the dark treatment (DARK), the highest HupA accumulation was observed during the first 12 weeks (**Figures 6**, **8**). Under the conditions associated with high HupA accumulation, i.e., under mixed light (RED–BLUE-L), the HupA content gradually increased up to 4.42 ± 0.48 mg·g^-1^ DW at 8 weeks and 4.31 ± 0.70 mg·g^-1^ DW at 12 weeks, followed by a gradual decrease to 2.85 ± 0.17 mg·g^-1^ DW at 24 weeks. Under blue light (BLUE-L) the HupA content decreased to 2.06 ± 0.99 mg·g^-1^ DW over the first six weeks and then increased until week 12 to reach 4.83 ± 0.21 mg·g^-1^ DW. Over the next six weeks the mean HupA content remained at a lower, but stable level of 2.52 ± 0.2 mg·g^-1^ DW. Under the white-light reference treatment (WHITE-L), HupA content increased until week 6 and then fluctuated within the range of 1.26 ± 0.01 mg·g^- 1^ DW at week 14 and 2.61 ± 0.70 mg·g^-1^ DW at week 24. Under the dark treatment (DARK), HupA content decreased from week 4 and showed the greatest fluctuations over the course of the experiment (**Figures 6**, **8**).

**Figure 8:**
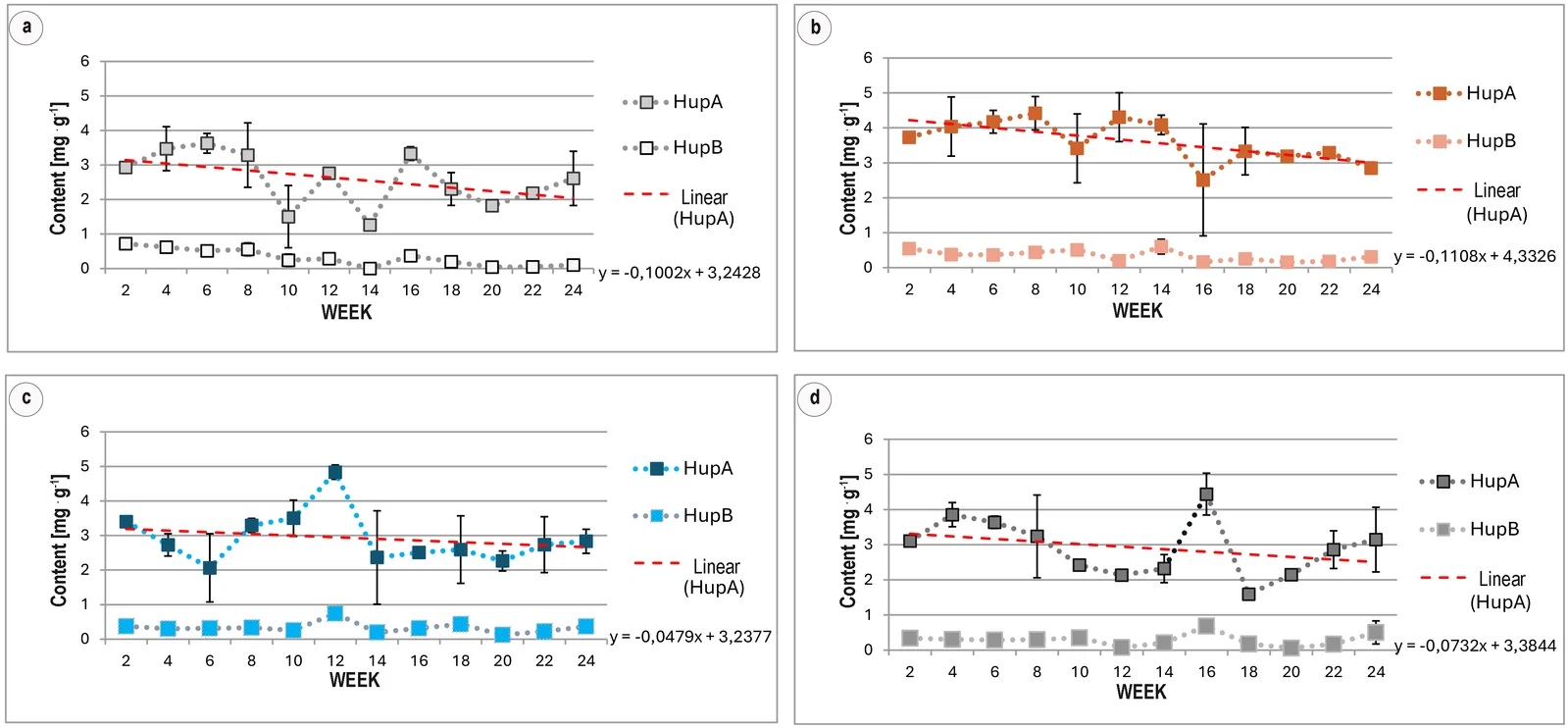
Time-course of huperzine A (HupA) and huperzine B (HupB) in *Huperzia selago* gametophyte extracts over 24 weeks under three light treatments and in darkness. Symbols show mean ± SD (n = 3 independent experiments; each value is the mean of 3 technical replicates). The red dashed line shows the least-squares linear fit to HupA, with the regression equation and R² reported in each panel. **(a)** white light (WHITE-L), **(b)** red/blue LEDs (RED–BLUE-L), **(c)** blue LEDs (BLUE-L), **(d)** darkness (DARK).

In all variants of the experiment, HupB concentrations remained low, within a narrow range of mean ≈ 0.05–0.25 mg·g^-^¹ DW, i.e. < 10% of simultaneously determined HupA concentration. The maximum HupB content was 0.74 ± 0.03 mg·g^-1^ DW gametophyte (12 weeks in culture, BLUE-L treatment). In some weeks under WHITE-L treatment, the HupB content fell below the limit of detection (14 weeks) or was very low (≤0.01 HupB mg·g^-^¹ DW). On average, the highest HupB content was observed in gametophytes cultured under red-blue light (RED–BLUE-L; 0.34 ± 0.16 mg·g^-^¹ DW), whereas the lowest mean value was recorded in darkness (DARK; 0.29 ± 0.18 mg·g^-^¹ DW).

### 3.3 Enzymatic assay results: acetylcholinesterase inhibition

The degree of AChE inhibition by *H. selago* gametophyte extracts is shown in **Figure 9**. Correlation and regression statistics for HupA content vs. AChE inhibition are provided in **Supplementary Table S2** (Pearson *r* with 95% CI, slope, *R*²) and **Supplementary Table S3** (Spearman ρ).

**Figure 9:**
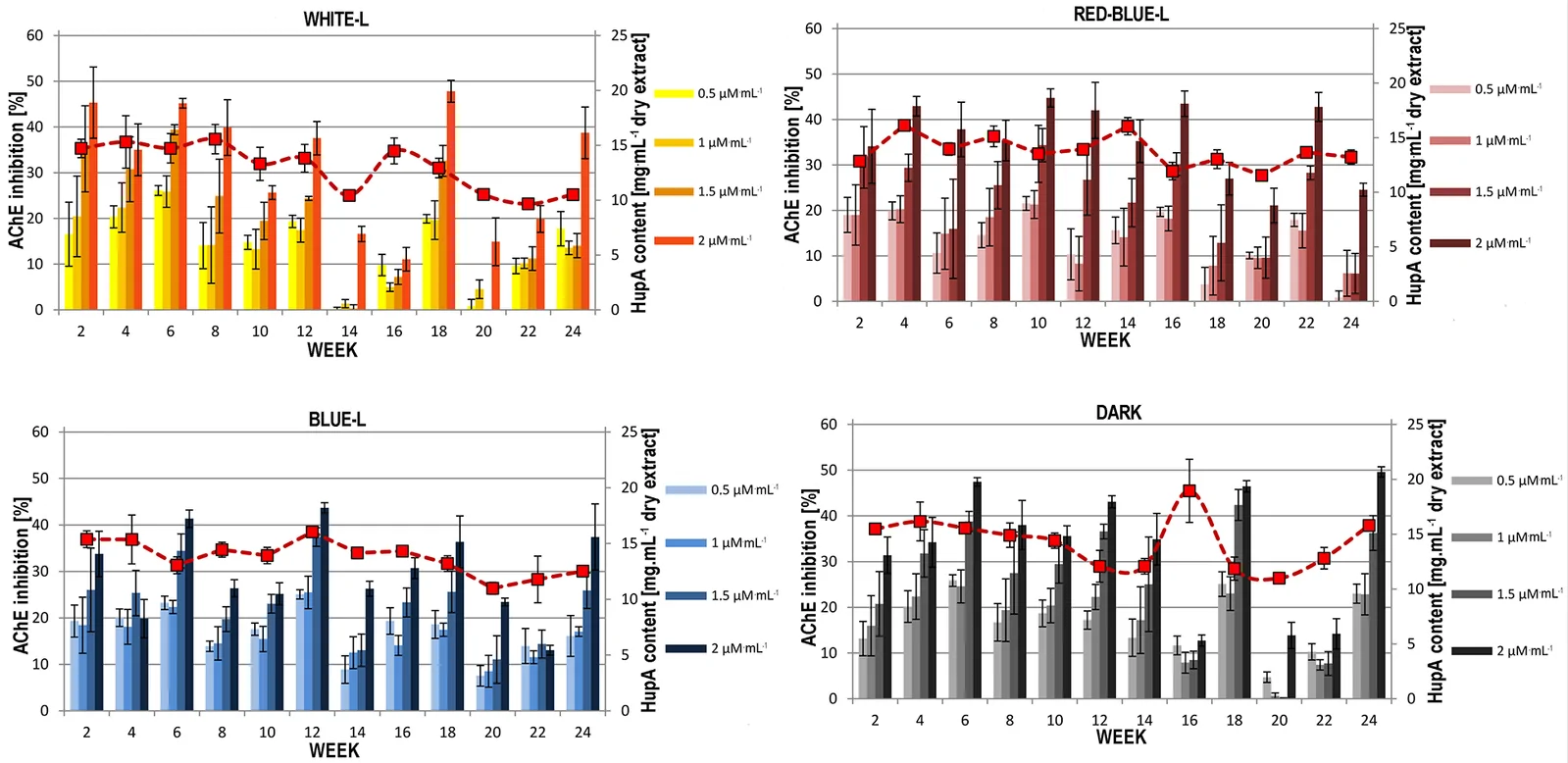
Acetylcholinesterase (AChE) inhibition by *Huperzia selago* gametophyte extracts collected over 24 weeks under three light treatments and in darkness, plotted together with the huperzine A (HupA) content of the corresponding extracts. Vertical bars show mean AChE inhibition (%) ± SD calculated from three independently prepared biological replicate extracts after averaging technical well readings within each biological replicate extract and concentration. Extracts were tested at four HupA-equivalent concentrations: 0.5, 1.0, 1.5 and 2.0 µM. The red dashed line (right-hand y-axis) shows HupA content per gram of dry extract (mg·g^-^¹). For statistical comparison among light treatments, replicate-level values were summarized to week × concentration means before applying the Kruskal–Wallis test. Correlation and regression analyses were performed using weekly mean AChE inhibition values after averaging technical replication and are provided in **Supplementary Tables S2, S3**.

The extracts were tested at four concentrations, corresponding to 0.5, 1.0, 1.5 and 2.0 µM HupA equivalents, and within that range the inhibition was up to 49.61 ± 1.12%. AChE inhibition by pure huperzine A (HupA), at the same concentrations, was 58.30 ± 8.86%, 55.37 ± 9.65%, 82.54 ± 4.68% and 80.05 ± 4.54%, respectively.

The mean AChE inhibition calculated across sampling times and extract concentrations was numerically highest for extracts obtained from gametophytes cultured in darkness (DARK; 23.13 ± 12.51%) and lowest for the white-light reference treatment (WHITE-L; 19.88 ± 12.75%). RED–BLUE-L and BLUE-L extracts showed very similar mean inhibition values, 21.69 ± 11.58% and 21.58 ± 8.72%, respectively. However, after averaging technical well readings within independently prepared biological replicate extracts and summarizing the data to the week × concentration level, differences among light treatments were not statistically significant (Kruskal–Wallis test, H(3, N = 192) = 1.87, p = 0.6006). Thus, no single light treatment produced a clearly dominant AChE-inhibitory response across the whole dataset.

Correlation analyses between HupA content and AChE inhibition were performed using weekly mean values after averaging technical well readings within biological replicate extracts. These analyses showed variable, generally weak-to-moderate relationships depending on light treatment and extract concentration. Pearson correlations were positive in several BLUE-L and WHITE-L combinations, but R² values remained low to moderate in most cases. Spearman rank correlations did not remain significant after FDR correction. Therefore, the correlation analyses indicate that HupA content alone did not fully explain the observed AChE inhibition, but they should not be interpreted as evidence of additive, synergistic or non-linear multi-component effects.

## 4 Discussion

Our experiments demonstrate that the spectral quality of light is an effective and easily controlled tool to modulate HupA and HupB accumulation in axenic *Huperzia selago* gametophyte cultures. Over the 24-week culture period, blue-light treatment (BLUE-L, λ = 450 nm) was associated with the strongest increase in HupA content, reaching 4.83 ± 0.21 mg·g^-^¹ DW at 12 weeks, whereas red–blue light treatment (RED–BLUE-L, λ = 660/450 nm) showed the highest mean HupA content over the whole culture period (3.61 ± 0.61 mg·g^-^¹ DW; p < 0.001) compared with the white-light reference treatment (WHITE-L, 2.59 ± 0.79 mg·g^-^¹ DW HupA). In contrast, the dark treatment promoted the strongest relative fresh-weight accumulation up to 24 weeks. This discrepancy between metabolite content per unit dry weight and relative fresh-weight accumulation indicates that the light treatment associated with the highest alkaloid content is not necessarily the same as the treatment promoting the strongest biomass increase. However, because biomass accumulation was assessed as a relative fresh-weight index and total dry biomass per culture vessel was not determined, the present study cannot directly calculate total HupA/HupB amount per vessel or productivity per unit time. Evaluation of changes in alkaloid accumulation during culture indicates a distinct HupA accumulation window in weeks 8–12, when the alkaloid level rapidly increased under blue-light and red–blue-light treatments. Under BLUE-L treatment, HupA content decreased over the first six weeks and then increased to its maximum at 12 weeks, followed by a plateau phase and then a slight decrease. Under RED–BLUE-L treatment, HupA levels increased to 4.42 ± 0.48 mg·g^-^¹ at 8 weeks and 4.31 ± 0.70 mg·g^-^¹ at 12 weeks and then decreased. The proposed 8–12-week harvest window was selected as a compromise between alkaloid accumulation and relative biomass increase. During this period, HupA content reached high values under RED–BLUE-L treatment at weeks 8 and 12 and the maximum value under BLUE-L treatment at week 12, while HupB also reached its maximum under BLUE-L at week 12. At the same time, relative fresh-weight accumulation was already substantial by weeks 8–12, especially in the dark treatment and under red–blue light. Prolonging the culture beyond this period increased maintenance time and operational costs, whereas HupA content tended to decrease or fluctuate after week 12. Therefore, the 8–12-week interval appears to be the most favorable harvest window based on the measured parameters, although direct productivity per vessel should be verified in future experiments using parallel dry-biomass measurements.

Huperzine B (HupB) was a minor metabolite under all treatments and typically its levels fluctuated within a narrow range of approximately 0.05–0.25 mg·g^-^¹ DW, i.e. <10% of simultaneously measured HupA levels. The maximum HupB content (0.74 ± 0.03 mg·g^-^¹ DW) was observed at 12 weeks under BLUE-L treatment while the highest average HupB levels (0.34 ± 0.16 mg·g^-^¹ DW) were obtained under RED–BLUE-L treatment, and the lowest (0.29 ± 0.18 mg·g^-^¹ DW) in darkness (DARK). Under the white-light reference treatment (WHITE-L), instances of HupB levels at the limit of detection (LoD) (e.g., week 14) or very low levels (≤0.01 mg·g^-^¹ DW) were observed. The measurements were confirmed using HPLC-DAD (*t*_R_ HupB in the standard ~11.08 min, in the extracts ~11.00 min) and LC–MS/IT–TOF (*t*_R_ 26.80 min; [M+H]^+^ *m*/*z* 257.1647, error <2 ppm). The results suggest that HupB accumulation responded to light treatment in a partly similar way to HupA, but HupB remained a minor metabolite and its net accumulation was much lower than that of HupA. At the molecular level, the above effects can be accounted for by the involvement of photoreceptors and light signal transduction pathways in the regulation of secondary metabolism. Blue light may activate cryptochromes and phototropins modulating the expression of genes involved in nitrogen metabolism, oxidative stress response and alkaloid biosynthesis which leads to a higher alkaloid content of dry mass (Peng et al., 2024; Szypuła and Pietrosiuk, 2025). In *H. selago* gametophytes, which develop very slowly in the wild, the presence of light and light-spectrum modifications are likely to affect the growth–alkaloid accumulation balance and favor secondary metabolite accumulation under blue and red–blue light, while darkness reduces photometabolic costs and promotes biomass accumulation with moderate alkaloid accumulation. Also, when alkaloid accumulation reaches high levels, the increasing gametophyte biomass may lead to a dilution effect expressed as lower metabolite content per unit dry weight.

However, it should be remembered that the physiology, biochemistry and genetics of Lycopodiaceae gametophytes, including *H. selago*, remain poorly understood. They may be monoecious, bulbous or cylindrical, with radial or bilateral symmetry. Subterranean gametophytes are achlorophyllous, long-lived (even several years), mycoheterotrophic and entirely dependent on mycorrhizal fungi while the epigeic (surface) types are green, faster growing and only partly rely on mycorrhiza (e.g. *Lycopodiella* Holub). Within the genus *Huperzia* gametophytes may have different anatomical features, but in the majority subterranean gametophytes predominate and *in vivo* their development is extremely slow. The development from a spore germinating in darkness to fertilization and the growth of a young sporophyte usually takes several years and the whole process requires the presence of endophytic fungi which form an evident layer within the prothallus (Pressel et al., 2016; Rimington et al., 2020). In the absence of early mycorrhiza, the prothallus stops developing and dies. These specific biological features justify the use of controlled *in vitro* cultures as a model as they make it possible to avoid the mycorrhizal barrier and dramatically shorten the time to the alkaloid-producing stage, which proved to be of key importance for the effect of light-dependent modulation of HupA/HupB accumulation observed in the current study.

*In vitro* cultures significantly accelerate the germination of spores and successive stages in the development of prothalli and mature gametophytes may be obtained within a few months, rather than years. In the wild, spores usually germinate after 2–3 years and gametophytes attain maturity after another 12–15 years (Freeberg and Wetmore, 1957; Whittier and Storchová, 2007; Szypuła et al., 2020; Szypuła, 2023). In the current study, we used stable cultures of prothalli obtained from spores, induced and maintained on Moore medium modified according to Whittier and Storchová (2007) and the gametophytes reached maturity after 6–12 months (Szypuła et al., 2020). This shortening of time to reach the stage of detectable alkaloid accumulation confirms the usefulness of gametophytes as a model for studying alkaloid accumulation. In the current study, as early as at 8 weeks in culture, high HupA accumulation was observed, with a mean content of 3.61 ± 0.61 mg·g^-^¹ DW and a maximum value of 4.83 mg·g^-^¹ DW, together with detectable HupB levels (up to 0.74 ± 0.03 mg·g^-^¹ DW, mean value 0.36 ± 0.05 mg·g^-1^ DW), which allows evaluating alkaloid accumulation in experiments lasting a few months. Also, the gametophytes demonstrated rapid biomass growth and a high viability. The biomass growth index at 24 weeks was approximately 2500% (FW) with 90–100% viability preserved in consecutive subcultures. Importantly, the growth rate of gametophytes was approximately 15-fold higher compared to sporophytes obtained from *H. selago* shoot fragments at 6 months in culture and nearly twice as high as that of sporophytes regenerated from somatic embryos, which additionally shortens the time needed to acquire sufficient biomass for phytochemical assays (Szypuła et al., 2005, 2020).

What is important from a practical perspective, spore-initiated gametophyte cultures are easier to maintain under axenic conditions than cultures initiated from sporophytes, which are usually associated with abundant endophytes and mycorrhizal microbiota. In addition, spore collection is non-destructive to wild populations, which is important from both conservation and logistical perspectives. Together with rapid biomass accumulation, high viability and early detectable HupA/HupB accumulation, these features make *H. selago* gametophyte cultures a valuable biotechnological model for studying alkaloid accumulation and for further optimization of controlled *in vitro* strategies aimed at obtaining Lycopodiaceae alkaloid-rich biomass. In addition, the axenic and developmentally uniform character of the culture system makes it suitable for future studies on metabolic regulation, elicitation and precursor feeding. Such a system may also support future metabolic engineering approaches once the key genes and regulatory steps of Lycopodiaceae alkaloid biosynthesis are better characterized.

The presented culture protocol potentially may offer a solution to the existing supply barrier facing plant raw materials of the Lycopodiaceae family. *Huperzia serrata*, a traditional raw material used to isolate the alkaloid HupA is extremely slow to grow (5–15 cm long sporophytes reach that length at the age of 10–20 years), entirely dependent on mycorrhizal symbioses and contains little HupA, usually 0.004–0.025% DW, while overexploitation of wild populations gives rise to serious conservation concerns (Ma et al., 2005, 2007). Field cultivation of Lycopodiaceae (club mosses) is impractical and a stable supply chain impossible to maintain without *in vitro* tissue cultures (Ma et al., 2005, 2007; Ferreira et al., 2016). In this situation, an *in vitro* system based on axenic *H. selago* gametophytes, using a reproducible and easily standardized protocol for HupA/HupB accumulation, with possible future transfer to photobioreactors, e.g., of the air-lift type, may allow metabolite-profile control at moderate operational costs (Szypuła and Pietrosiuk, 2021a; Pérez Aguilar et al., 2025). Comparison of HupA content values indicates that this approach provides high alkaloid levels per unit dry weight compared with several previously reported culture systems. In *H. serrata* sporophytes, HupA levels are typically reported as 0.004–0.025% DW, which corresponds to 0.04–0.25 mg·g^-^¹ DW while in other *Huperzia* Bernh. species reported to contain HupA, levels rarely exceed approximately 1–2 mg·g^-^¹ DW (*H. pinifolia* ~1.7 mg·g^-^¹ DW) (Lim et al., 2010; Ishiuchi et al., 2013). For *H. selago* collected in the wild HupA levels of up to ~1.59 mg·g^-^¹ DW have been reported; in sporophytes regenerated from somatic embryos up to ~3.33 mg·g^-^¹ DW and in gametophyte cultures up to ~4.73 mg·g^-^¹ DW (Szypuła et al., 2005, 2011, 2020).

Compared to other types of *in vitro* cultures of club mosses, the HupA levels obtained in the current study in *H. selago* gametophytes (mean: 2.6–3.6 mg·g^-^¹ DW under red–blue light treatment; maximum: 4.83 mg·g^-^¹ DW under BLUE-L treatment) are evidently above the HupA levels so far reported for the genus *Phlegmariurus* Holub. In *P. squarrosus* the HupA levels in callus and/or prothallium tissue usually ranged from ~0.3 to 0.7 mg·g^-^¹ DW depending on the type of culture, solid or suspension) (Ma and Gang, 2008). In the recently reported suspension cultures of *P. taxifolius* the HupA content was only 2.0–2.5 µg·g^-^¹ DW (~0.002–0.0025 mg·g^-^¹ DW), even after a transfer to an airlift bioreactor (Pérez Aguilar et al., 2025). Such differences in HupA content among *in vitro* systems suggest that the selection of the plant development stage and tissue type — gametophytes vs. sporophyte-derived tissues (organs, callus or suspension cultures) and the ambient factors, such as LED spectral control in the current study may evidently modulate HupA accumulation and its content in dry biomass. Axenic gametophytes of *H. selago* therefore appear to offer a promising balance between high HupA content per unit dry weight and stable axenic growth. However, direct comparison of total alkaloid productivity between culture systems requires data on total dry biomass, total alkaloid amount per culture vessel and culture duration.

A limitation of the present study is that biomass accumulation in the light-spectrum experiment was expressed as a relative fresh-weight index, whereas HupA and HupB were quantified as content per gram of dry weight. In our previous work on *H. selago* gametophyte cultures, biomass increase was assessed both on a fresh-weight and dry-weight basis, showing that these parameters may follow broadly similar but not identical trends during culture development (Szypuła et al., 2020). However, in the present experiment, total dry biomass per culture vessel was not determined for each light treatment and sampling time. Therefore, the present data allow comparison of HupA/HupB content in dry biomass and relative fresh-weight accumulation, but they do not allow direct calculation of total alkaloid amount per culture vessel or productivity per unit time. For this reason, the treatment effect should be interpreted as modulation of alkaloid accumulation per unit dry weight and relative biomass increase, not as direct maximization of total alkaloid production. Future optimization should include parallel measurements of fresh and dry biomass, total HupA/HupB amount per vessel and productivity expressed per unit time. Future transfer of this system to photobioreactors will require additional optimization. Although LED lighting provides precise spectral control, large-scale cultivation of slowly developing gametophyte biomass may be limited by light penetration, vessel geometry, biomass density, aeration or mixing conditions, maintenance of axenic cultures and the operational costs of long-term illumination. Therefore, before scale-up, the proposed culture strategy should be validated in larger vessels with simultaneous monitoring of fresh and dry biomass, HupA/HupB content, total alkaloid amount per vessel and energy-related culture costs.

Extracts obtained from *H. selago* gametophytes revealed evident, though moderate, inhibition of AChE activity at standardized concentrations. The inhibitory response varied with extract concentration and sampling time, but after averaging technical well readings within independently prepared biological replicate extracts and summarizing the data to the week × concentration level, no statistically significant differences among light treatments were detected. Therefore, the AChE results should not be interpreted as indicating one clearly superior light treatment for enzyme inhibition.

The extracts were standardized to HupA equivalents, and the HupA content of dry extracts remained relatively similar among light treatments. Nevertheless, the relationship between HupA content and AChE inhibition was variable and generally weak-to-moderate. This indicates that HupA content alone did not fully explain the observed biological activity of the extracts. A possible contribution of HupB, other co-occurring Lycopodiaceae alkaloids and matrix-related factors cannot be excluded, but this requires further confirmation by bioassay-guided fractionation, testing of isolated compounds and/or defined alkaloid mixtures. Therefore, the present data should be treated as indicating a possible multi-component contribution, not as direct evidence of additive or synergistic effects.

This interpretation is consistent with previous reports on club moss extracts, where AChE inhibition was not always proportional to HupA content alone and where other Lycopodiaceae alkaloids, including HupB, 6*β*-hydroxyhuperzine A and lycodine-like derivatives, were also considered relevant for the biological activity of the extracts (Ortega et al., 2004; Garcia et al., 2017; Jiang et al., 2024; Le et al., 2025).

According to the literature, at least 13 alkaloids have been found in the tissues of *H. selago* sporophytes (Achmatowicz and Rodewald, 1955, 1956; Rodewald and Grynkiewicz, 1968; Ayer et al., 1989; Ma and Gang, 2004; Staerk et al., 2004; Czapski et al., 2014; Szypuła and Pietrosiuk, 2021b). Of these, in addition to HupA, inhibitory activity against AChE has been confirmed for such alkaloids as huperzine B, 6β-hydroxyhuperzine A and *N*-methylhuperzine B, also isolated from *H. serrata*. According to Wu and Gu (2006), they may enhance the AChE-inhibitory activity of huperzine A. *H. selago* prothalli obtained *in vitro* remain the only haploidal club moss structures which have been thoroughly screened for the presence of alkaloids (Szypuła et al., 2020). In addition to huperzine A and huperzine B, a broader group of Lycopodiaceae alkaloid features has been detected and putatively annotated, including compounds related to deacetylfawcettine, fawcettimine, 16-hydroxyhuperzine B, deacetyllycoclavine, annopodine, lycopecurine, des-*N*-methylfastigiatine and flabelline. In the present study, this alkaloid profile was further supported by accurate-mass measurement, molecular-formula assignment and, for several features, MS/MS fragmentation acquired in the same experimental material. The compounds listed include representatives of at least three classes of skeleton alkaloids specific to the Lycopodiaceae family, namely lycodine-, lycopodine- and fawcettimine-type alkaloids, which suggests the co-occurrence of different alkaloid skeletons in one type of tissue. In view of earlier reports of at least 13 *H. selago* alkaloids, including acrifoline, 12-epilycodoline, lycodoline, lycopodine, selagoline, serratinidine, 6β-hydroxyhuperzine A, α-/β-obscurine, HupB, serratine and lucidoline, the findings we present considerably increase knowledge of the chemical diversity of club mosses and demonstrate that gametophytes may produce a diversified spectrum of alkaloid features, some of which have not been routinely reported from sporophyte tissues.

Also, the biological activity of the extract may depend not only on the level of one marker alkaloid, that is HupA, but also on the broader alkaloid profile. This supports the use of bioassay-guided fractionation in further research to screen the extracts for active alkaloid fractions, determine the chemical structures of relevant compounds and evaluate their biological activity, including determination of the half-maximal inhibitory concentration (IC_50_).

However, because most non-target alkaloids in the current LC–MS/IT–TOF profile were assigned as putative annotations or isomeric candidate sets, their definitive structural confirmation will require isolation, authentic standards and/or NMR analysis. In addition, the extraction parameters themselves can influence the degree of AChE inhibition, occasionally resulting in its stronger inhibition despite lower measured HupA levels in simple methanolic extracts. This inconsistency is most likely due to differences in the extraction efficiency and stability of co-occurring cationic alkaloids. Because their protonation state and resulting salt forms strongly influence solubility in methanolic media and availability to the enzymatic reaction, extracts with lower quantified HupA content may nevertheless exhibit stronger AChE inhibition. Thus, the standardization of the extracts aimed at enhancing their activity should also involve optimization of the extraction, rather than solely maximizing HupA content (Dymek et al., 2021; Dymek and Mroczek, 2021).

Interestingly, many currently marketed dietary supplements labeled to contain huperzine A in fact contain extracts of *Huperzia serrata*, not pure HupA. In such products the HupA content is declared as a marker of quality, and the co-occurring alkaloids such as HupB, 6β-hydroxyhuperzine A or lycodine-type derivatives are not determined and listed in the composition, although, as suggested by our findings, they may influence the overall AChE-inhibitory activity of the extract. As a result, it may often be impossible to compare the potency between batches of one supplement and between products with a declared identical HupA content but a different qualitative alkaloid profile or matrix. That is why standardization should involve comprehensive chemical profiling, including HPLC-DAD/LC–MS fingerprinting, quantitative analysis of at least two or three major alkaloids, and functional evaluation, such as percentage AChE inhibition at a reference dose or the IC_50_ of the fraction/extract. Such an approach limits batch-to-batch variability and better reflects the actual biological activity of the extract. As seen in the model we propose, the HupA content alone does not fully account for the potency of AChE inhibition by the extract.

In summary, we have demonstrated that LED spectral control, especially using blue and red-blue light, is effective in modulating HupA and HupB accumulation in axenic *Huperzia selago* gametophytes. Blue and red-blue LEDs were associated with the highest HupA contents within the 8–12-week harvest window, whereas darkness promoted the strongest relative fresh-weight accumulation. HupB remained a minor but light-responsive metabolite. In the context of the unique biological features of gametophytes and the impracticality of club moss field cultivation, the established *in vitro* model offers a promising and ethically sourced system for obtaining Lycopodiaceae alkaloid-rich biomass under controlled conditions, and showed HupA contents comparable to or higher than those reported for many sporophyte-based *in vivo* and *in vitro* systems.

For potential larger-scale applications, the results suggest a promising strategy for future testing rather than an already validated culture protocol. The continuous DARK treatment promoted biomass accumulation, whereas continuous BLUE-L and RED–BLUE-L treatments were associated with higher HupA/HupB accumulation during the 8–12-week harvest window. Therefore, a sequential approach consisting of biomass accumulation in darkness followed by a short induction phase under blue or red–blue light may be considered for future experiments. However, such a two-stage regime was not directly tested in the present study and requires experimental validation before it can be recommended for cultivation. Future experiments should also include monochromatic red light, far-red light and red/far-red transitions. These treatments would allow separation of general LED-mediated effects on HupA/HupB accumulation from more specific phytochrome-related regulation, which is particularly relevant in the context of possible links between Lycopodiaceae alkaloids, AChE-inhibitory activity and light-dependent signaling.

## 5 Conclusions

The current study confirms that the spectral quality of light is a powerful, controllable tool to modulate HupA and HupB accumulation, as reflected by treatment-dependent differences in alkaloid content in axenic gametophyte cultures of *Huperzia selago*. Blue-light treatment (λ ≈ 450 nm) was associated with the strongest increase in HupA content, reaching 4.83 ± 0.21 mg·g^-^¹ DW at 12 weeks, whereas red–blue light treatment (660/450 nm) maintained the highest mean HupA content over the 24-week culture period (3.61 ± 0.61 mg·g^-^¹ DW), and darkness promoted relative biomass accumulation. Together, these results suggest a tentative two-stage strategy for future validation, consisting of biomass accumulation in darkness followed by induction under blue or red–blue light within the 8–12-week harvest window. Huperzine B (HupB) remained a minor but light-responsive metabolite (≤ 0.74 ± 0.03 mg·g^-^¹ DW). The extracts significantly inhibited AChE at low doses; the variable relationship between HupA content and AChE inhibition suggests that HupA content alone may not fully explain the biological activity of the extracts. Gametophyte cultures offer rapid biomass growth, stable axenic conditions and non-destructive sourcing, and thus represent a promising, eco-friendly and operationally sound biotechnological system for obtaining HupA/HupB-containing biomass and standardized alkaloid-rich extracts under controlled *in vitro* conditions. The results provide factual data on process control, including light spectrum and harvest window, and quality control based on HPLC-DAD, LC–MS/IT–TOF and MS/MS-supported alkaloid profiling. The current study supports the role of *H. selago* gametophytes as a controlled and reproducible *in vitro* system for Lycopodiaceae alkaloid accumulation, while addressing key supply constraints.

## Data Availability

The original contributions presented in the study are included in the article/**Supplementary Material**. Further inquiries can be directed to the corresponding author.
