# Supplementary material for: Light spectrum effects on huperzine A and B accumulation in axenic gametophyte cultures of Huperzia selago (L.) Bernh. ex Schrank et Mart
Source: Front Plant Sci. 2026 Oct 5;17:1933008. doi: 10.3389/fpls.2026.1933008 (PMC13677926; doi:10.3389/fpls.2026.1933008)
Supplement: Supplementary file 1 [file DataSheet1.docx]

**Supplementary Figure S1.** Representative HPLC–DAD chromatograms of authentic huperzine B (HupB) and huperzine A (HupA) standards and a representative Huperzia selago gametophyte extract.
(A) Chromatogram of authentic HupB and HupA standards.
(B) Chromatogram of a representative *H. selago* gametophyte extract obtained in the experiment. Detection was performed at 308 nm under the chromatographic conditions described in Section 2.4.3. Insets show UV spectra recorded at the peak apex for HupA and HupB. Peaks in the extract were identified by comparison of retention behavior and UV spectra with the authentic standards. Panels are shown with different y-axis scaling to improve peak visibility.
