# Supplementary material for: Light spectrum effects on huperzine A and B accumulation in axenic gametophyte cultures of Huperzia selago (L.) Bernh. ex Schrank et Mart
Source: Front Plant Sci. 2026 Oct 5;17:1933008. doi: 10.3389/fpls.2026.1933008 (PMC13677926; doi:10.3389/fpls.2026.1933008)
Supplement: Supplementary file 2 [file Table1.docx]

**Supplementary Table S1. Alkaloids detected in *Huperzia selago* gametophyte extracts by LC–MS/IT–TOF in positive-ion ESI mode. Putative annotations are based on accurate-mass measurement, molecular-formula prediction, and, where available, MS/MS fragmentation acquired in the present study. Huperzine A and huperzine B were confirmed by comparison with authentic standards in HPLC–DAD analysis and additionally supported by accurate mass and MS/MS fragmentation.**

| Peak ID | Ion* | m/z (exp.) | m/z (calcd.) | Mass error (signed Δppm) | Formula | Putative annotation (primary) | Alternative candidates | Alkaloid class/skeleton | MSI level | Evidence** | MS/MS fragments (m/z) | Notes |
| --- | --- | --- | --- | --- | --- | --- | --- | --- | --- | --- | --- | --- |
| 1 | [M+H]⁺ | 264.1962 | 264.1958 | +1.51 | C_16_H_25_NO_2_ | Fawcettimine | fawcettimine-type alkaloid | fawcettimine-type | 3 | MS1; MS/MS | 264, 246, 228, 213, 188, 172, 150, 136, 122, 105, 91 | Accurate mass is consistent with fawcettimine, and the observed MS/MS spectrum shows partial overlap with published fawcettimine fragmentation, including the product ions at m/z 246 and 150 (Dymek et al., 2021). The assignment is supported at the fawcettimine-type level, but remains putative in the absence of an authentic standard or reference spectrum acquired under identical conditions. |
| 2 | [M+H]⁺ | 266.2109 | 266.2115 | -2.25 | C_16_H_27_NO_2_ | Deacetylfawcettine | — | fawcettimine-type | 3 | MS1; MS/MS | 266, 248, 230, 202, 176 | Accurate-mass/formula match; MS/MS acquired and consistent with a fawcettimine-type feature. Assignment remains putative without an authentic standard/reference spectrum. |
| 3 | [M+H]⁺ | 278.1754 | 278.1751 | +1.08 | C_16_H_23_NO_3_ | 4α,6α-dihydroxyanhydrolycodoline-related isomer | 4α,6α-dihydroxyanhydrolycodoline; Lycoposerramine E; Huperzine O; 4α-hydroxyserratidine; 6α-hydroxyserratidine; dihydroannotine | huperzine/lycodine-type | 3 | MS1; MS/MS | 278, 260, 146, 113 | Accurate mass and MS/MS fragmentation support an oxygenated lycodine-type alkaloid. The observed product ions overlap more closely with published fragmentation of 4α,6α-dihydroxyanhydrolycodoline (Dymek et al., 2021) than with dihydroannotine, but the assignment remains tentative because several isomeric Lycopodiaceae alkaloids are plausible and only partial fragment overlap was observed. |
| 4 | [M+H]⁺ | 264.1947 | 264.1958 | -4.16 | C_16_H_25_NO_2_ | Lycoposerramine L/M-related isomer | Lycoposerramine L/M; Lucidoline; Lycodoline; Lycoposerramine J | lycopodine-type | 3 | MS1; MS/MS | 264, 246, 188, 150, 122 | Accurate mass and MS/MS fragmentation support a C_16_H_25_NO_2_ lycopodine-type alkaloid. The observed product ions are compatible with reported fragmentation of lycoposerramine L/M-related features (Dymek et al., 2021), but isomer-level discrimination remains impossible without authentic standards or reference spectra. |
| 5 | [M+H]⁺ | 280.1903 | 280.1907 | -1.43 | C_16_H_25_NO_3_ | Hydroxylated lycopodine-type alkaloid | Dihydroxylycopodine isomer | lycopodine-type | 3 | MS1; MS/MS | 280, 262, 244, 216, 188, 174, 150, 91 | Accurate mass and MS/MS fragmentation support a hydroxylated lycopodine-type alkaloid. The observed product ions partly overlap with published fragmentation of 8β,11α-dihydroxylycopodine (Dymek et al., 2021), but the assignment remains tentative because isomeric Lycopodiaceae alkaloids are possible and not all diagnostic fragments were observed. |
| 6 | [M+H]⁺ | 259.1805 | 259.1805 | +0.04 | C_16_H_22_N_2_O | 11-hydroxylycodine-related isomer | des-*N*-methyl-β-obscurine | huperzine/lycodine-type | 3 | MS1; MS/MS | 259, 227, 210, 190, 180, 167, 165, 152, 130, 91 | Accurate mass and MS/MS fragmentation support a C_16_H_22_N_2_O lycodine-type alkaloid. 11-Hydroxylycodine is the most plausible candidate, whereas *N*-methyl dihydrolycodine was excluded based on exact-mass mismatch. Des-*N*-methyl-β-obscurine was retained only as a tentative alternative candidate because the observed fragmentation shows limited overlap with published data. |
| 7 | [M+H]⁺ | 273.1599 | 273.1598 | +0.37 | C_16_H_20_N_2_O_2_ | Hydroxylated huperzine/lycodine-type alkaloid | 16-hydroxyhuperzine B–related isomer | lycodine-type | 3 | MS1 | — | Accurate mass is consistent with a hydroxylated huperzine/lycodine-type alkaloid. Literature MS/MS data make 16-hydroxyhuperzine B a plausible candidate (Dymek et al., 2021), but the precursor-ion intensity was too low to acquire a reliable MS/MS spectrum in the present study; therefore, the assignment remains tentative. |
| 8 | [M+H]⁺ | 262.1800 | 262.1802 | -0.76 | C_16_H_23_NO_2_ | Lycoposerramine K-related isomer | Huperzine I; huperzinine; lycoposerramine C/H/I/K isomers; serratidine | huperzine/lycodine-type | 3 | MS1; MS/MS | 262, 244, 189, 160, 105 | Accurate mass and MS/MS fragmentation are more consistent with a lycoposerramine K-type alkaloid than with serratidine. The observed product ions, especially m/z 244, 189, 160 and 105, show the closest overlap with published lycoposerramine K fragmentation (Dymek et al., 2021), but the assignment remains tentative because isomeric C_16_H_23_NO_2_ Lycopodiaceae alkaloids are possible. |
| 9 | [M+H]⁺ | 266.2109 | 266.2115 | -2.25 | C_16_H_27_NO_2_ | C_16_H_27_NO_2_ lycopodine-/fawcettimine-type isomer | deacetyllycoclavine; deacetylfawcettine; C16H27NO2 isomer | lycopodine-/fawcettimine-related isomer | 3 | MS1 | — | A second chromatographically distinct C_16_H_27_NO_2_ feature was detected. The precursor-ion intensity was too low to acquire a reliable MS/MS spectrum; therefore, assignment remains tentative and isomeric ambiguity cannot be excluded. |
| 10 | [M+H]⁺ | 257.1642 | 257.1648 | -2.33 | C_16_H_2_0N_2_O | Huperzine B | — | huperzine/lycodine-type | 1 | MS1; MS/MS; HPLC-DAD; Std | 257, 240, 212, 198, 184, 158, 154, 130, 115, 91 | Confirmed as HupB based on accurate mass, MS/MS fragmentation and agreement with an authentic standard in HPLC-DAD. Published fragmentation of huperzine B shows the closest overlap with the observed spectrum (Dymek et al., 2021). |
| 11 | [M+H]⁺ | 243.1481 | 243.1492 | -4.52 | C_15_H_18_N_2_O | Huperzine A | — | huperzine/lycodine-type | 1 | MS1; MS/MS; HPLC-DAD; Std | 243, 227, 210, 196, 192, 178, 165, 154, 115, 91 | Confirmed as HupA by agreement with an authentic standard in HPLC-DAD and MS/MS fragmentation consistent with HupA. |
| 12 | [M+H]⁺ | 305.2217 | 305.2224 | -2.29 | C_18_H_28_N_2_O_2_ | Serratinidine | — | lycopodine-type | 3 | MS1; MS/MS | 305, 264, 246, 228 | Accurate-mass/formula match; MS/MS acquired. Annotation remains putative without a standard/reference spectrum. |
| 13 | [M+H]⁺ | 292.1901 | 292.1907 | -2.05 | C_17_H_25_NO_3_ | Annopodine / Lyconnotine (isomeric assignment) | — | lycopodine-type | 3 | MS1; MS/MS | 292, 274, 218, 186, 174, 162, 158, 147 | Accurate-mass match; MS/MS acquired. Isomeric ambiguity between annopodine and lyconnotine cannot be fully excluded without an authentic standard/reference spectrum. |
| 14 | [M+H]⁺ | 248.1998 | 248.2009 | -4.48 | C_16_H_25_NO | Lycopodine / selagoline-type alkaloid | Lycopodine / Selagoline | lycopodine-type | 2 | MS1; MS/MS | 248, 230, 200, 188, 174, 145, 131, 105, 91 | Accurate mass and MS/MS fragmentation support a lycopodine/selagoline-type alkaloid. The observed product ions overlap with published fragmentation reported for both lycopodine and selagoline (Dymek et al., 2021), and the assignment therefore remains tentative at the isomer level. |
| 15 | [M+H]⁺ | 250.2156 | 250.2165 | -3.60 | C_16_H_27_NO | C_16_H_27_NO lycopodine-type alkaloid | lycopecurine; dihydrolycopodine | lycopodine-type | 3 | MS1 | — | Accurate mass is consistent with a C_16_H_27_NO lycopodine-type alkaloid. Literature MS/MS data indicate dihydrolycopodine as a plausible candidate (Dymek et al., 2021), but the precursor-ion intensity was too low to acquire a reliable MS/MS spectrum in the present study; therefore, structure-level assignment remains tentative. |
| 16 | [M+H]⁺ | 287.2113 | 287.2118 | -1.74 | C_18_H_26_N_2_O | Des_-_*N*-methylfastigiatine | — | fastigiatine-type | 3 | MS1 | — | This was a low-intensity feature, and the precursor-ion intensity was too low to acquire a reliable MS/MS spectrum. Annotation is therefore based on accurate-mass/formula match and literature data. |
| 17 | [M+H]⁺ | 289.2275 | 289.2274 | +0.35 | C_18_H_29_N_2_O | Flabelline | — | Lycopodiaceae alkaloid | 3 | MS1; MS/MS | 289, 230, 146, 105 | Accurate mass is fully consistent with flabelline, and the observed MS/MS spectrum shows partial overlap with published fragmentation, including the characteristic product ion at m/z 230 (Dymek et al., 2021). However, several reported product ions were not observed; therefore, the assignment remains putative in the absence of an authentic standard or reference spectrum acquired under identical conditions. |
| 18 | [M+H]⁺ | 276.1950 | 276.1958 | -2.90 | C_17_H_25_NO_2_ | Lycoflexine | Magellanine; epilobscurinol; α-/β-obscurine isomers | fawcettimine-type | 3 | MS1 | — | Accurate-mass match supports annotation as a C_17_H_25_NO_2_ Lycopodiaceae alkaloid, with lycoflexine as a plausible candidate. The precursor-ion intensity was too low to acquire a reliable MS/MS spectrum; therefore, related isomers cannot be excluded. |

Notes: m/z (calcd.) refers to the monoisotopic protonated molecule [M+H]⁺; mass error is reported as signed Δppm.

Evidence codes: MS1, accurate-mass measurement and molecular-formula assignment; MS/MS, tandem mass spectrum acquired in the present study; HPLC–DAD, photodiode-array detection; Std, authentic standard used for target-compound confirmation.

MSI confidence: Level 1, compound confirmed by comparison with an authentic standard; Level 2, putatively identified compound or isomer set supported by characteristic MS/MS fragmentation; Level 3, putative annotation or compound class based on accurate-mass/formula match, with or without non-diagnostic MS/MS data; isomers possible.

Abbreviations: ESI, electrospray ionization; LC–MS/IT–TOF, liquid chromatography coupled with ion-trap time-of-flight mass spectrometry; MSI, Metabolomics Standards Initiative; ppm, parts per million

Literature references cited in the Notes column refer to previously reported Lycopodiaceae alkaloids and/or their MS/MS fragmentation patterns used for comparison with the spectra acquired in the present study.
