# Supplementary material for: Light spectrum effects on huperzine A and B accumulation in axenic gametophyte cultures of Huperzia selago (L.) Bernh. ex Schrank et Mart
Source: Front Plant Sci. 2026 Oct 5;17:1933008. doi: 10.3389/fpls.2026.1933008 (PMC13677926; doi:10.3389/fpls.2026.1933008)
Supplement: Supplementary file 3 [file Table2.docx]

**Supplementary Table S2** **Correlations between HupA content (mg·g⁻¹ dry extract) and AChE inhibition (%) across light regimes and extract concentrations after averaging technical well readings within independently prepared biological replicate extracts and summarizing the data to weekly means.**

Pearson r [95% CI], two-sided p, significance; linear model: R² and slope = Δ%AChE per 1 mg·g⁻¹ HupA.

| **Regime** | **Concentration (µM)** | **n** | **Pearson r** | **95% CI** | **p** | **Unadj. Sig.** | **R²** | **Slope**  **(% per mg·g⁻¹)** |
| --- | --- | --- | --- | --- | --- | --- | --- | --- |
| BLUE-L | 0.5 | 12 | 0.585 | [0.017, 0.868] | 0.0455 | * | 0.343 | 2.021 |
| BLUE-L | 1.0 | 12 | 0.623 | [0.077, 0.882] | 0.0304 | * | 0.388 | 1.904 |
| BLUE-L | 1.5 | 12 | 0.549 | [-0.037, 0.854] | 0.0646 | n.s. | 0.301 | 2.871 |
| BLUE-L | 2.0 | 12 | 0.314 | [-0.317, 0.753] | 0.3196 | n.s. | 0.099 | 1.883 |
| DARK | 0.5 | 12 | 0.215 | [-0.409, 0.702] | 0.5015 | n.s. | 0.046 | 0.587 |
| DARK | 1.0 | 12 | 0.143 | [-0.469, 0.663] | 0.6575 | n.s. | 0.020 | 0.470 |
| DARK | 1.5 | 12 | 0.021 | [-0.560, 0.588] | 0.9495 | n.s. | 0.000 | 0.120 |
| DARK | 2.0 | 12 | -0.045 | [-0.604, 0.543] | 0.8888 | n.s. | 0.002 | -0.258 |
| RED–BLUE-L | 0.5 | 12 | 0.222 | [-0.403, 0.706] | 0.4882 | n.s. | 0.049 | 1.025 |
| RED–BLUE-L | 1.0 | 12 | 0.298 | [-0.333, 0.744] | 0.3473 | n.s. | 0.089 | 1.095 |
| RED–BLUE-L | 1.5 | 12 | 0.249 | [-0.379, 0.720] | 0.4353 | n.s. | 0.062 | 1.618 |
| RED–BLUE-L | 2.0 | 12 | 0.367 | [-0.262, 0.777] | 0.2407 | n.s. | 0.135 | 2.043 |
| WHITE-L | 0.5 | 12 | 0.602 | [0.044, 0.874] | 0.0382 | * | 0.363 | 2.191 |
| WHITE-L | 1.0 | 12 | 0.607 | [0.051, 0.876] | 0.0363 | * | 0.369 | 2.158 |
| WHITE-L | 1.5 | 12 | 0.706 | [0.223, 0.911] | 0.0103 | * | 0.499 | 4.418 |
| WHITE-L | 2.0 | 12 | 0.478 | [-0.132, 0.826] | 0.1158 | n.s. | 0.229 | 2.922 |

Notes: For AChE inhibition, three independently prepared biological replicate extracts were used for each light treatment and sampling week. Technical well readings were first averaged within each biological replicate extract and concentration; biological replicate means were then summarized to obtain weekly mean values for each light regime and extract concentration. Therefore, each correlation was calculated using weekly mean values for a given light regime and extract concentration (n = 12).

Significance stars refer to unadjusted p-values only (*p* < 0.05; **p* < 0.01; ***p* < 0.001; n.s., not significant). Pearson’s *r* was calculated with two-sided *p* values; 95% CI was calculated via Fisher’s *z* transformation. Linear model: %AChE inhibition = β₀ + β₁·HupA; R² from OLS; slope = β₁. The correlations should be interpreted as exploratory and do not demonstrate additive or synergistic effects.
