# Supplementary material for: Light spectrum effects on huperzine A and B accumulation in axenic gametophyte cultures of Huperzia selago (L.) Bernh. ex Schrank et Mart
Source: Front Plant Sci. 2026 Oct 5;17:1933008. doi: 10.3389/fpls.2026.1933008 (PMC13677926; doi:10.3389/fpls.2026.1933008)
Supplement: Supplementary file 4 [file Table3.docx]

**Supplementary Table S3. Spearman’s ρ between HupA content (mg·g⁻¹ dry extract) and AChE inhibition (%) by light regime and extract concentration after averaging technical well readings within independently prepared biological replicate extracts and summarizing the data to weekly means.**

Values shown: Spearman’s ρ, two-sided p, Benjamini–Hochberg FDR q across 16 tests, and significance coding based on unadjusted p.

| **Regime** | **Concentration (µM)** | **n** | **Spearman ρ** | **p-value** | **FDR q** | **Unadjusted Sig.** |
| --- | --- | --- | --- | --- | --- | --- |
| BLUE-L | 0.5 | 12 | 0.54 | 0.0709 | 0.2561 | n.s. |
| BLUE-L | 1.0 | 12 | 0.57 | 0.0548 | 0.2561 | n.s. |
| BLUE-L | 1.5 | 12 | 0.44 | 0.1517 | 0.4046 | n.s. |
| BLUE-L | 2.0 | 12 | 0.27 | 0.4038 | 0.6312 | n.s. |
| DARK | 0.5 | 12 | 0.23 | 0.4697 | 0.6312 | n.s. |
| DARK | 1.0 | 12 | 0.21 | 0.5049 | 0.6312 | n.s. |
| DARK | 1.5 | 12 | 0.07 | 0.8372 | 0.9204 | n.s. |
| DARK | 2.0 | 12 | -0.01 | 0.9741 | 0.9741 | n.s. |
| RED–BLUE-L | 0.5 | 12 | 0.21 | 0.5128 | 0.6312 | n.s. |
| RED–BLUE-L | 1.0 | 12 | 0.22 | 0.4845 | 0.6312 | n.s. |
| RED–BLUE-L | 1.5 | 12 | 0.06 | 0.8629 | 0.9204 | n.s. |
| RED–BLUE-L | 2.0 | 12 | 0.33 | 0.2969 | 0.5938 | n.s. |
| WHITE-L | 0.5 | 12 | 0.52 | 0.0800 | 0.2561 | n.s. |
| WHITE-L | 1.0 | 12 | 0.66 | 0.0185 | 0.1763 | * |
| WHITE-L | 1.5 | 12 | 0.65 | 0.0220 | 0.1763 | * |
| WHITE-L | 2.0 | 12 | 0.38 | 0.2170 | 0.4960 | n.s. |

**Notes:** Spearman’s rank correlations were calculated within each light regime and extract concentration using weekly mean AChE inhibition values. For each light treatment and sampling week, AChE inhibition was assessed using three independently prepared biological replicate extracts. Technical well readings were first averaged within each biological replicate extract and concentration; biological replicate means were then summarized to weekly mean values used for correlation analysis (n = 12 per row). FDR q-values were obtained using the Benjamini–Hochberg procedure across the 16 correlations. Significance stars refer to unadjusted p-values only (*p* < 0.05; **p* < 0.01; ***p* < 0.001; n.s., not significant). After FDR correction, none of the Spearman correlations remained significant at q < 0.05. These exploratory correlations do not demonstrate additive or synergistic effects.
